# Chromosome-level genome assembly of *Zizania latifolia* provides insights into its seed shattering and phytocassane biosynthesis

**DOI:** 10.1038/s42003-021-02993-3

**Published:** 2022-01-11

**Authors:** Ning Yan, Ting Yang, Xiu-Ting Yu, Lian-Guang Shang, De-Ping Guo, Yu Zhang, Lin Meng, Qian-Qian Qi, Ya-Li Li, Yong-Mei Du, Xin-Min Liu, Xiao-Long Yuan, Peng Qin, Jie Qiu, Qian Qian, Zhong-Feng Zhang

**Affiliations:** 1 Tobacco Research Institute of Chinese Academy of Agricultural Sciences, Qingdao, 266101 China; 2 Graduate School of Chinese Academy of Agricultural Sciences, Beijing, 100081 China; 3 Shenzhen Branch, Guangdong Laboratory of Lingnan Modern Agriculture, Genome Analysis Laboratory of the Ministry of Agriculture and Rural Affairs, Agricultural Genomics Institute at Shenzhen, Chinese Academy of Agricultural Sciences, Shenzhen, 518120 China; 4 Department of Horticulture, College of Agriculture and Biotechnology, Zhejiang University, Hangzhou, 310058 China; 5 State Key Laboratory of Crop Gene Exploration and Utilization in Southwest China, Rice Research Institute, Sichuan Agricultural University, Chengdu, Sichuan 611130 China; 6 Shanghai Key Laboratory of Plant Molecular Sciences, College of Life Sciences, Shanghai Normal University, Shanghai, 200234 China; 7 State Key Laboratory of Rice Biology, China National Rice Research Institute, Chinese Academy of Agricultural Sciences, Hangzhou, 310006 China

**Keywords:** Comparative genomics, Comparative genomics

## Abstract

Chinese wild rice (*Zizania latifolia*; family: Gramineae) is a valuable medicinal homologous grain in East and Southeast Asia. Here, using Nanopore sequencing and Hi-C scaffolding, we generated a 547.38 Mb chromosome-level genome assembly comprising 332 contigs and 164 scaffolds (contig N50 = 4.48 Mb; scaffold N50 = 32.79 Mb). The genome harbors 38,852 genes, with 52.89% of the genome comprising repetitive sequences. Phylogenetic analyses revealed close relation of *Z. latifolia* to *Leersia perrieri* and *Oryza* species, with a divergence time of 19.7–31.0 million years. Collinearity and transcriptome analyses revealed candidate genes related to seed shattering, providing basic information on abscission layer formation and degradation in *Z. latifolia*. Moreover, two genomic blocks in the *Z. latifolia* genome showed good synteny with the rice phytocassane biosynthetic gene cluster. The updated genome will support future studies on the genetic improvement of Chinese wild rice and comparative analyses between *Z. latifolia* and other plants.

## Abstract (web summary)

Yan, Yang, Yu, Shang et al. provide a chromosome-level reference genome for Chinese wild rice (*Zizania latifolia*) along with phylogenetic and transcriptomic analyses. Their results identify candidate genes related to seed shattering and synteny with the rice phytocassane biosynthetic gene cluster.

## Introduction

Chinese wild rice (*Zizania latifolia*) is a diploid (2*n* = 2× = 34), perennial, and aquatic grass belonging to the tribe *Oryzeae* Dum, family Gramineae^1–3^. *Z. latifolia* originated in China and is distributed in China, Korea, Japan, and Southeast Asian countries^1,2^. Chinese wild rice is one of the earliest important cereal crops in China and has been consumed as a cereal for more than 3000 years^4^. Since the Tang Dynasty, Chinese wild rice has been used as a traditional medicine food homologous grain^1,4^. A medical book written in the Ming dynasty, Compendium of Materia Medica, recorded the use of Chinese wild rice for adjuvant treatment of diabetes and gastrointestinal diseases^1^. The health-promoting effects of Chinese wild rice include atherosclerosis prevention, alleviation of lipotoxicity, and insulin resistance^3,5–7^. In China, *Z. latifolia* infected by the endophytic *Ustilago esculenta* has been domesticated as the second-largest aquatic vegetable, known as ‘*Jiaobai*’^8–13^. Therefore, *Z. latifolia* is an important economic crop with high nutritional and medicinal value, and worthy of further investigation.

*Z. latifolia* is usually grown in Asia, but *Zizania palustris*, *Zizania aquatica*, and *Zizania texana* are commonly found in North America^2,14,15^. The perennial *Z. latifolia* and annual *Z. palustris* are used for the commercial production of Chinese and northern wild rice, respectively^2^. Owing to long-term adaptation to environmental changes and resistance to abiotic and biotic stresses, genetic variation and useful gene resources of wild relatives of rice (e.g., *Z. latifolia*) have formed and accumulated during evolution^16^. Notably, *Z. latifolia* has several excellent traits not found in rice, including high protein content, high biomass productivity, deep-water tolerance, and blast resistance^1,3,17^. The protein, dietary fiber, and total phenolic concentrations in Chinese wild rice are ~2-, ~5-, and ~6-fold of those in rice, respectively^1,3^. Thus, *Z. latifolia* represents a potential gene donor for overcoming the bottleneck of narrow genetic resources in rice breeding^1,3,17–19^.

Seed shattering is an important trait for wild rice to adapt to the natural environment and maintain population reproduction^20,21^. The loss of seed shattering is a key event in rice domestication^22^. Because of the successful selection of varieties with low seed shattering^23^, the commercial production of northern wild rice has been realized in the United States. As a type of health food with a unique flavor, high nutritional value, and high price, northern wild rice has entered people’s diets, and is also exported to China and Europe.

Biosynthetic gene clusters are critical genetic factors for the rapid environmental adaptability of plants^24,25^. Two biosynthetic gene clusters are well characterized in the rice genome: the phytocassane biosynthetic gene cluster on chromosome 2^26^ and the momilactone biosynthetic gene cluster on chromosome 4^27^. The potential functions of both gene clusters involve defense against pathogens and weeds in rice^26,28,29^. Additionally, the momilactone biosynthetic gene cluster is involved in the biosynthesis of allelochemicals^30^. However, it is not known whether the synthetic genes of phytocassane and momilactone are clustered in the genome of *Z. latifolia*.

Within the genus *Zizania*, the *Z. latifolia* genome was first sequenced in 2015 using next-generation sequencing technology^31^. The *Z. latifolia* genome completed by Guo et al.^31^ has been used for transcriptome analyses of the possible molecular mechanism of swollen culm formation in *Z. latifolia* induced by *U. esculenta*^8,9^. Because of technical limitations and a lack of a linkage map, the previous genome was only assembled at the scaffold level and remained relatively fragmented, with a contig N50 of 14 kb^31^. In this study, we generated the Chinese wild rice genome using a combination of Nanopore and Illumina sequencing data sets. Genome sequences of ~547.38 Mb were assembled with a contig N50 of 4.78 Mb and placed into 17 pseudochromosomes assisted by Hi-C. The updated and improved genome facilitated the annotation of protein-coding genes and noncoding RNAs. In this study, collinearity and transcriptome analyses revealed candidate genes involved in abscission layer formation (ALF) and degradation (ALD). Additionally, the phytocassane biosynthetic gene cluster was identified in the *Z. latifolia* genome, with complementary subclusters separating in two chromosomes. The updated *Z. latifolia* genome sequence serves as an important resource for comparative genomic studies between the Gramineae family and other plant species and might facilitate the rapid domestication of Chinese wild rice.

## Results

### Genome assembly, anchoring, and quality evaluation

**Fig. 1:**
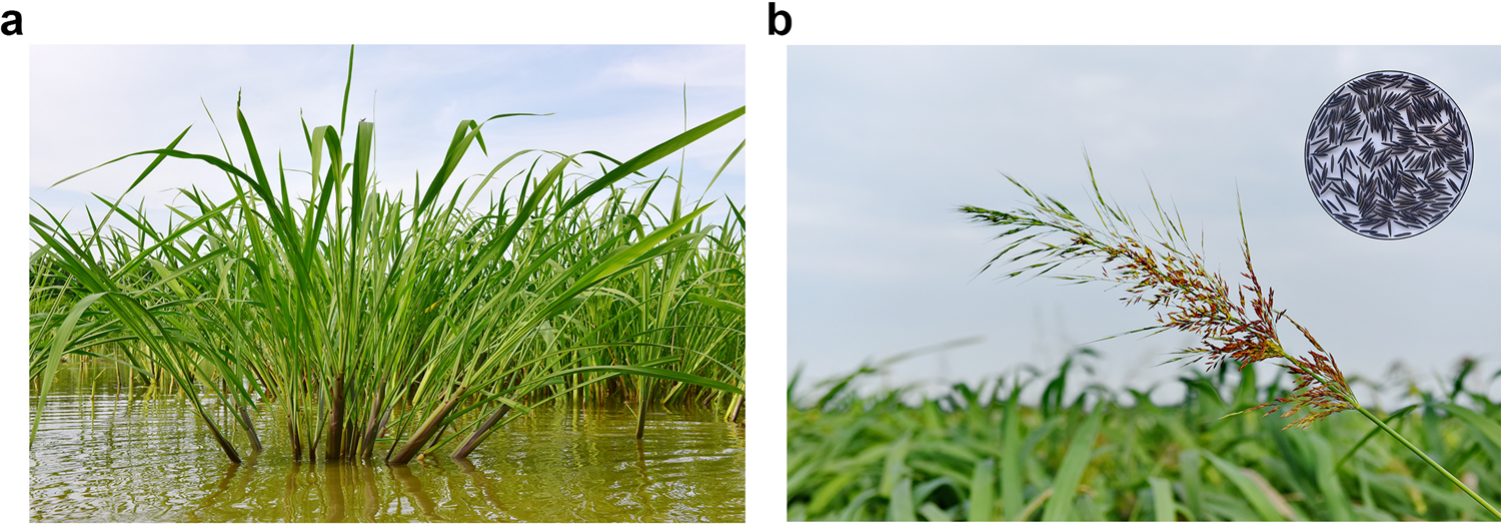
Photographs of Chinese wild rice plants and inflorescence with seeds. **a** Chinese wild rice plants growing in a paddy field. **b** Inflorescence and seed morphology of Chinese wild rice. The pictures were taken by Ning Yan from Tobacco Research Institute of Chinese Academy of Agricultural Sciences.

**Fig. 2:**
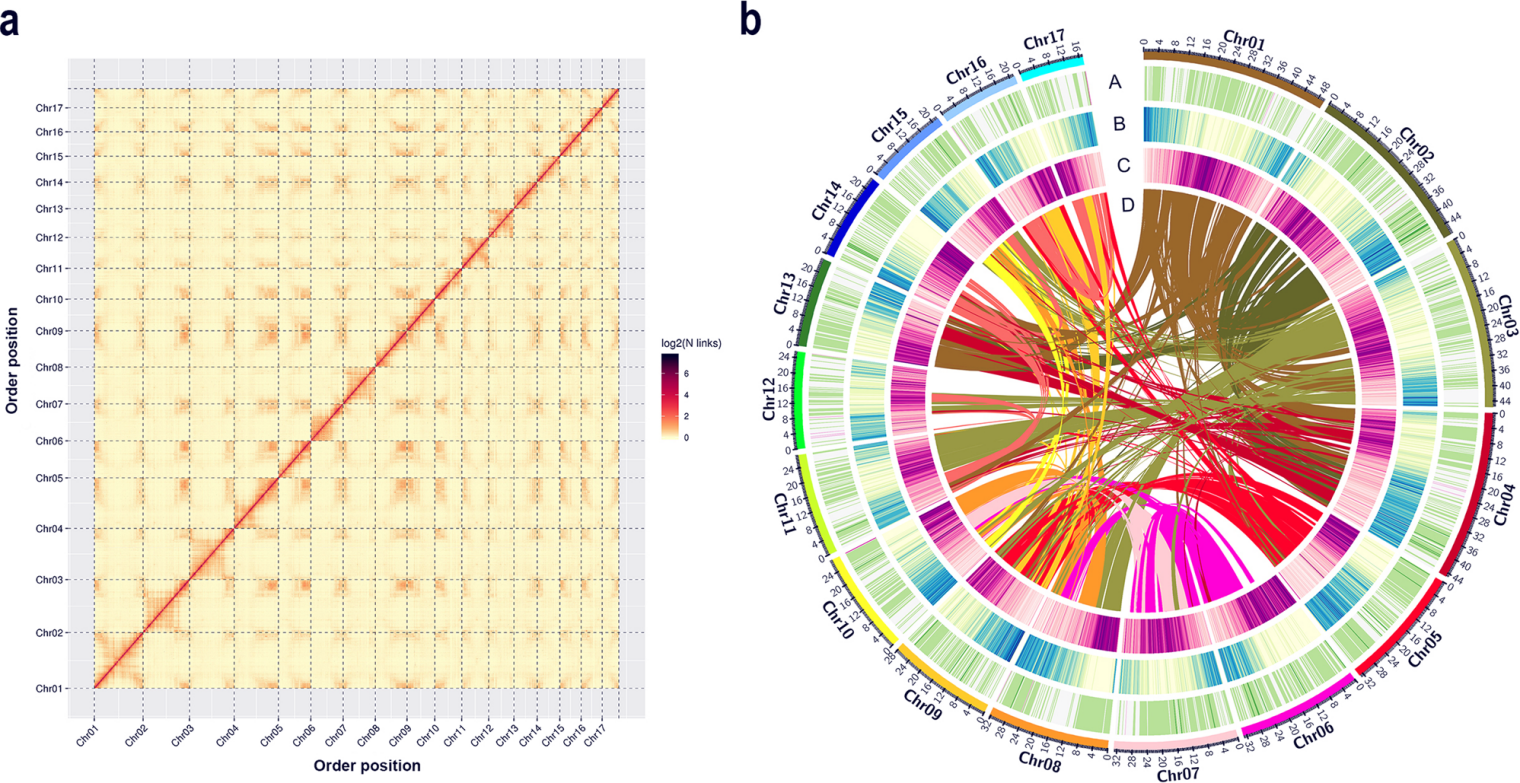
Chinese wild rice genome information. **a** Hi-C contact data mapped on the updated Chinese wild rice genome showing genome-wide all-by-all interactions. **b** Overview of the Chinese wild rice genome. Ring A: distribution of GC content (green); ring B: gene density (blue); ring C: density of repeat sequences (purple); and ring D: syntenic blocks within the genome.

In this study, we sampled Chinese wild rice plants grown in a paddy field (Fig. 1a). The inflorescence of Chinese wild rice is a panicle with multiple branches (Fig. 1b); we found both male and female flowers on the same branch, with the female flower above the male flower. The seeds of Chinese wild rice were blackish brown, cylindrical, and tapered at both ends. One *Z. latifolia* plant (accession Huai’an) was selected for whole-genome sequencing, and two paired-end Illumina libraries were constructed and sequenced. After cleaning, 68.50 Gb of high-quality sequencing data were obtained. Genome characterization based on *K*-mer depth distribution revealed that the Chinese wild rice Huai’an genome size was ~606.13 MB, with 49.00% repeats, 0.18% heterozygosity, and 42.88% GC content. Subsequently, we constructed and sequenced a Nanopore library, and 61.56 Gb of high-quality sequencing data were obtained, representing ~112.46× of the Chinese wild rice genome. The detailed summary statistics of the Oxford Nanopore Technology and Illumina sequencing are provided in Supplementary Table 1. After correction with Illumina sequencing and Hi-C scaffolding, we generated an assembly of 547.38 Mb comprising 332 contigs and 164 scaffolds, with a contig N50 of 4.48 Mb and a scaffold N50 of 32.79 Mb (Table 1). Based on the Hi-C interaction maps, 300 sequences covering ~545.36 Mb were clustered into 17 groups that corresponded to the 17 chromosomes of Chinese wild rice (Fig. 2), with the shortest being 17.01 Mb and the longest being 49.61 Mb (Table 1, Supplementary Table 2).

**Table 1:** Sequencing and assembly statistics of the Chinese wild rice genome.

Sequencing and assembly	Number	Size	N50 length
Nanopore reads	3,970,614	61.56 Gb	20.43 kb
Final assembly (contigs)	332	547.38 Mb	4.48 Mb
Final assembly (scaffolds)	164	547.40 Mb	32.79 Mb
Chromosome-anchored contigs	300	545.36 Mb	–

The Illumina reads were mapped to the assembled genome using the Burrows–Wheeler Alignment software^32^ to assess the quality of the genome assembly. Evaluation using CEGMA v2.5^33^ with a database of 458 clusters of essential genes (CEGs) and 248 highly conserved CEGs indicated that 98.25% (450) and 94.76% (235) of the CEGs and highly conserved CEGs were present in the Chinese wild rice genome assembly, respectively. Further evaluation using Benchmarking Universal Single-Copy Orthologues (BUSCO) indicated that 97.71% (1,577) of the core genes were complete in the Chinese wild rice genome assembly, including single copies (77.39%, 1,249) and duplicated copies (20.32%, 328) (Supplementary Table 3). Additionally, 0.56% (9) of the core genes were fragmented, and only 1.73% (28) were missing. The BUSCO-based method for evaluating genome assembly integrity suggested that our Chinese wild rice Huai’an genome assembly shows better assembly integrity (Supplementary Table 4) than the previously assembled Chinese wild rice HSD2 genome^31^. This result was further supported by the long terminal repeat (LTR) assembly index (LAI)-based method^34^ for evaluating genome assembly (Supplementary Table 5), which indicated that our Chinese wild rice Huai’an genome assembly showed an improved quality (LAI = 13.57) relative to the previously assembled Chinese wild rice HSD2 genome (LAI = 6.88)^31^ (Supplementary Table 5).

### Genome annotation

Genome annotation resulted in the identification of 289.56 Mb (52.89%) of repetitive sequences in the assembled genome, which is substantially greater than that in the previous assembled version (227.50 Mb [37.70%] of repetitive sequences)^31^. The predominant repetitive sequences were LTR retrotransposons, which constituted 37.58% of the Chinese wild rice genome assembly (Supplementary Table 6). Among the transposable element (TE) superfamily studied, Copia (22.78%) and Gypsy (12.50%) generally occupied a relatively high proportion of the Chinese wild rice genome, whereas the Polinton superfamily, which is a unique TE type of *Z. latifolia*, only accounted for a small proportion (Supplementary Table 6). Moreover, the repetitive rate in the genome of Chinese wild rice Huai’an (52.89%) is higher than that in the *Oryza sativa japonica* (40.43%) and *Oryza sativa indica* (42.05%) groups^35^. These results might explain why Chinese wild rice has a genome larger than *O. sativa*. Notably, the repetitive rate in the genome of northern wild rice (76.40%)^36^ is higher than that in Chinese wild rice Huai’an (52.89%), which might explain why northern wild rice has a larger genome than Chinese wild rice. We then used three strategies (ab initio prediction, a homology-based strategy, and transcriptomic support) to predict the protein-coding genes (Supplementary Fig. 1). Finally, 38,852 protein-coding genes (136.02 Mb) were obtained (Table 2) and annotated (Supplementary Data 1). Further comparisons between northern and Chinese wild rice revealed two genomes with differential protein-coding genes, 46,491 in northern wild rice and 38,852 in Chinese wild rice. Of these predicted genes, 36,473 (93.88%) were annotated using eight functional databases. Additionally, we identified 149 microRNAs (miRNAs), 397 rRNAs, 723 tRNAs, and 1368 pseudogenes (Table 2).

**Table 2:** Genome annotation statistics of the Chinese wild rice genome.

Genome annotation	Number	Size	Percentage (%)
Pseudogenes	1,368	4.55 Mb	0.83
miRNAs	149	–	–
rRNAs	397	–	–
tRNAs	723	–	–
Total protein-coding genes	38,852	136.02 Mb	24.85

### Evolution of the Chinese wild rice genome

**Fig. 3:**
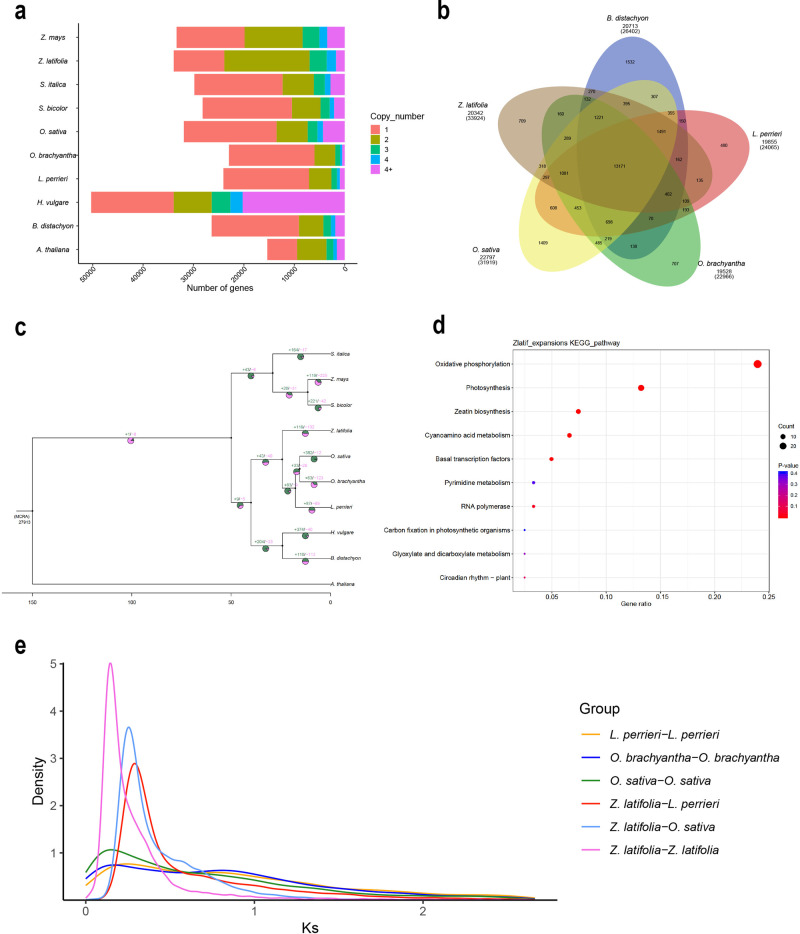
Comparative genomic analyses of Chinese wild rice genome. **a** Distribution of gene copy number in Chinese wild rice and nine other species. **b** Venn diagram of shared orthologous gene families in Chinese wild rice and other four related gramineous species (*Brachypodium distachyon*, *Leersia perrieri*, *Oryza brachyantha*, and *O. sativa*). **c** Phylogenetic tree of Chinese wild rice and nine other species. “+” represents the number of gene families expanded on the node and “-” represents the number of gene families contracted on the node. The pie chart shows the proportion of the corresponding branch contraction and expansion gene families. **d**, KEGG enrichment analyses for the expanded genes in the Chinese wild rice genome. **e** Ks distribution in Chinese wild rice and other representative species.

We performed comparative genomic analysis of the Chinese wild rice genome with the genome sequences of representative plant species, including eight gramineous plants (*Brachypodium distachyon*, *Hordeum vulgare*, *Leersia perrieri*, *Oryza brachyantha*, *O. sativa*, *Sorghum bicolor*, *Setaria italica*, and *Zea mays*) and one dicotyledon (*Arabidopsis thaliana*) clustered into 38,169 gene families. In total, 33,924 gene families were identified in the Chinese wild rice genome, 310 of which were specific to Chinese wild rice (Supplementary Fig. 2, Supplementary Table 7). Gene family analysis revealed that the single-copy genes in Chinese wild rice accounted for 25.82% of the predicted genes, which was substantially lower than that in other gramineous species (Fig. 3a). In contrast, a higher proportion of gene families with two copies was observed in the *Z. latifolia* genome, which could be explained by a recent whole-genome duplication (WGD) event. The clustering of gene families in Chinese wild rice and the other four gramineous species (*B. distachyon*, *L. perrieri*, *O. brachyantha*, and *O. sativa*) indicated that 13,171 gene families are shared among the five grass species and that they could be the core gene families (Fig. 3b). In total, 709 gene families were specific to *Z. latifolia*, which is similar to that of *O. brachyantha*.

Based on 1,371 single-copy genes in Chinese wild rice and nine other plant species, we constructed a phylogenetic tree (Supplementary Fig. 3), which showed that *Z. latifolia* is relatively closely related to *L. perrieri*, *O. sativa*, and *O. brachyantha*. *Z. latifolia* diverged from *L. perrieri* around 19.7 Mya to 31.0 Mya, which was before the divergence of *O. sativa* and *O. brachyantha* (14.0–16.0 Mya) (Supplementary Fig. 3). In Chinese wild rice, 119 gene families showed expansion, whereas 132 gene families exhibited contraction (Fig. 3c). The Kyoto Encyclopedia of Genes and Genomes (KEGG) pathway analysis indicated that genes related to oxidative phosphorylation, photosynthesis, zeatin biosynthesis, and cyanoamino acid metabolism were enriched in the expanded Chinese wild rice gene families (Fig. 3d). The strong positive section of genes is of great significance to the generation of new functions. The selection of positive genes in *Z. latifolia* is shown in Supplementary Data 2. Moreover, the KEGG pathway analysis indicated that the selection of positive genes was mainly related to carbon metabolism, biosynthesis of amino acids, and peroxisome (Supplementary Fig. 4).

WGD events are associated with an ancient polyploidization event predating the divergence of cereals^37^ and are of great significance for understanding gene neofunctionalization and genome evolution^38^. This study showed that recent WGD events occurred in the *Z. latifolia* genome after splitting from *O. sativa* (Fig. 3e), which is consistent with the previous findings^31^. *Zizania*–*Oryza* speciation events have both led to an increase in the genome size of northern wild rice (1.29 Gb)^36^ in comparison with that of rice (390.30 Mb) and Chinese wild rice (547.38 Mb) (Table 1). Moreover, a peak centering on Ks of ~0.25 was observed between the *O. sativa* and *Z. latifolia* pairs.

### Collinearity between genomes and seed-shattering-related genes of *O. sativa* and *Z. latifolia*

Significant genome collinearity has been observed between northern wild rice and rice^36,39,40^. Similarly, in this study, significant genome collinearity was observed between Chinese wild rice and rice (Supplementary Fig. 5a). To the best of our knowledge, 10 seed-shattering-related genes have been identified in rice (i.e., *qSH1*, *OsGRF4/PT2*, *OsSh1*, *OsNPC1*, *sh4/SHA1*, *SHAT*, *OsLG1*, *SH5*, *sh-h/OsCPL1*, and *SSH1*) (Supplementary Table 8). Moreover, 17 seed-shattering-related genes have been identified in northern wild rice through collinearity between seed-shattering-related genes of rice and northern wild rice^36^. Based on genome collinearity and gene homolog analyses between the genomes of Chinese wild rice and rice, we identified 29 candidate genes potentially related to seed shattering in Chinese wild rice (Supplementary Fig. 5b, Supplementary Data 3). In Chinese wild rice, collinearity was observed for two (*ZlqSH1a* and *ZlqSH1b*), seven (*ZlGRF4/PT2a*, *ZlGRF4/PT2b*, *ZlGRF4/PT2c*, *ZlGRF4/PT2d*, *ZlGRF4/PT2e*, *ZlGRF4/PT2f*, and *ZlGRF4/PT2g*), two (*ZlSh1a* and *ZlSh1b*), two (*ZlNPC1a* and *ZlNPC1b*), two (*ZlSHATa* and *ZlSHATb*), four (*ZlLG1a*, *ZlLG1b*, *ZlLG1c*, and *ZlLG1d*), two (*Zlsh4/SHA1a* and *Zlsh4/SHA1b*), two (*ZlSH5a* and *ZlSH5b*), two (*Zlsh-h/ZlCPL1a* and *Zlsh-h/ZlCPL1b*), and four (*ZlSSH1a*, *ZlSSH1b*, *ZlSSH1c*, and *ZlSSH1d*) genes with *qSH1*, *OsGRF4/PT2*, *OsSh1*, *OsNPC1*, *SHAT*, *OsLG1*, *sh4/SHA1*, *SH5*, *sh-h/OsCPL1*, and *SSH1* in rice, respectively (Supplementary Fig. 5b, Supplementary Data 3). According to their position in the evolutionary tree, the seed-shattering candidate genes of *Z. latifolia* were divided into four categories (Supplementary Fig. 6). Moreover, the motifs of these proteins were similar in the same group and different in different groups, which confirmed the reliability of grouping (Supplementary Fig. 7). The protein sequence alignment of seed-shattering genes in *Z. latifolia* and *Oryza* species is shown in Supplementary Figs. 8–17. Notably, aligning a sequence of seed-shattering candidates across *Z. latifolia* and *Oryza* species would help to identify the potential functional polymorphism and specific candidate sites for editing in *Z. latifolia*.

### Histologic, transcriptome, and phytohormone analyses of ALF and ALD tissues in Chinese wild rice

**Fig. 4:**
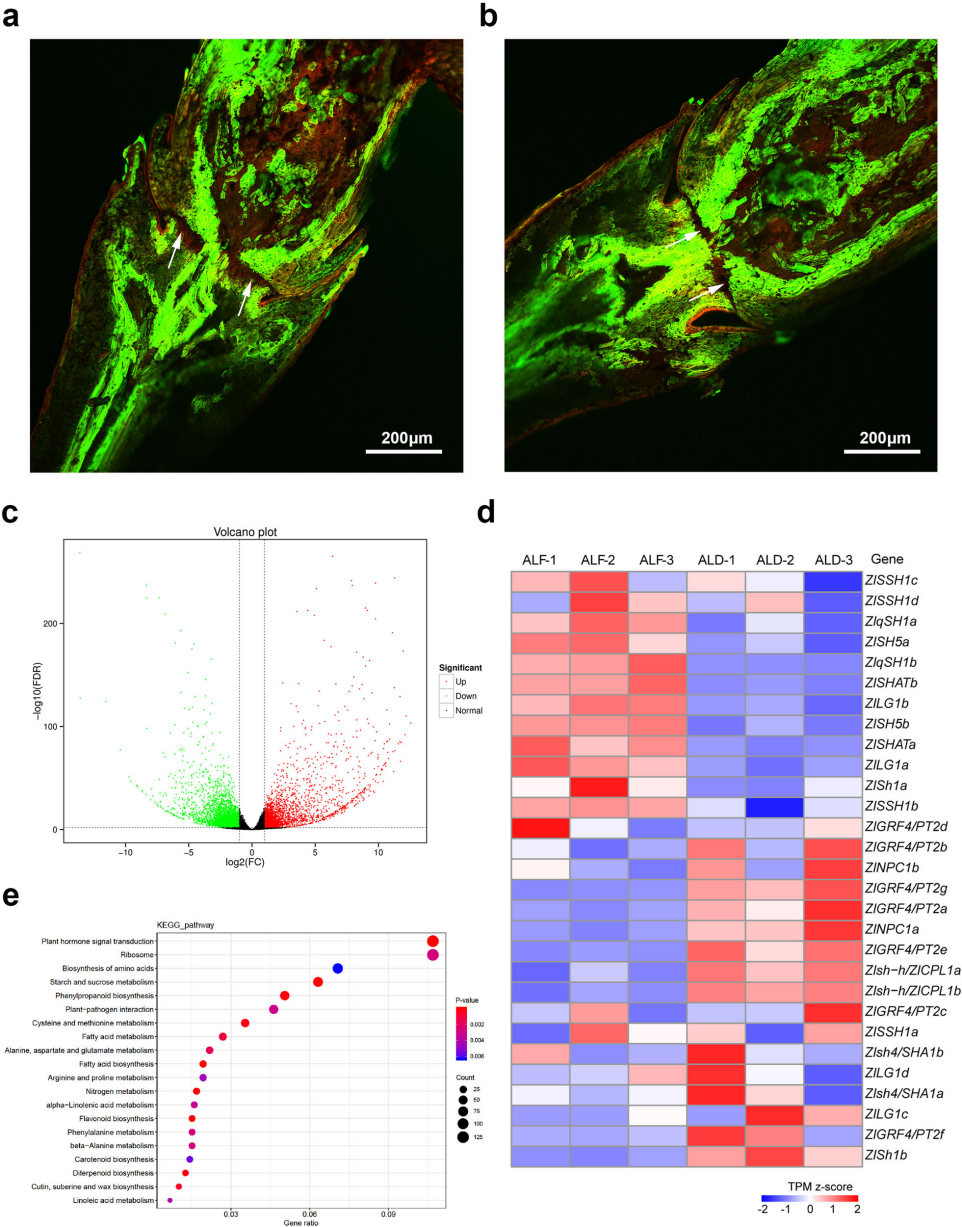
Histologic and transcriptome analyses of abscission layer formation (ALF) and degradation (ALD) in Chinese wild rice. **a** ALF and **b**, ALD as revealed by staining with the cell-permeant dye Acridine Orange (green fluorescence: dye bound to dsDNA; red fluorescence: dye bound to ssDNA or RNA). The white arrows indicate the abscission layer. Scale bar = 200 μm. **c** Volcano plot of differentially expressed genes between ALF and ALD. In this figure, green, red, and black dots represent genes with a low expression, high expression, and non-differentially expressed genes, respectively. **d** Expression levels of genes related to seed shattering between ALF and ALD. **e** KEGG enrichment bubble plot of differentially expressed genes between ALF and ALD.

During seed abscission, one or several layers of parenchyma cells differentiate from the abscission zone to form the abscission layer. Cells in the adjacent cell layer have thick, lignified cell walls, which help provide the mechanical force needed for abscission^41^. The results of the histological analysis showed that the abscission layer of Chinese wild rice comprises 6–8 circles of cells radially distributed in the periphery, where the single cells were oval, demonstrating a compact and regular arrangement that could be stained red by the cell-permeant dye, Acridine Orange (Fig. 4a, b). Moreover, we observed that the ALF and ALD of Chinese wild rice were complete, and that ALD led to seed shattering (Fig. 4a, b). To elucidate the potential molecular mechanism of seed shattering in Chinese wild rice, we analyzed abscission tissues from ALF and ALD by transcriptome sequencing. Compared with ALF, ALD involved 2,827 upregulated and 3,938 downregulated genes, including nine genes related to seed shattering (Fig. 4c, Supplementary Data 4). Among them, *ZlGRF4/PT2a* (*Zla08G018880*) and *ZlGRF4/PT2g* (*Zla05G008960*) were upregulated in ALD, whereas *ZlqSH1b* (*Zla02G027130*), *ZlSHATa* (*Zla07G002730*), *ZlSHATb* (*Zla06G014800*), *ZlLG1a* (*Zla07G002370*), *ZlLG1b* (*Zla06G015090*), *ZlSH5a* (*Zla01G006060*), and *ZlSH5b* (*Zla13G006450*) were downregulated (Fig. 4d, Supplementary Data 4). To confirm the reliability of the transcriptome results, the expression levels of eight of these key genes were further examined using real-time PCR (qRT-PCR) evaluation. *ZlqSH1b*, *ZlSHATa*, *ZlSHATb*, *ZlLG1a*, *ZlLG1b*, *ZlSH5a*, and *ZlSH5b* expression was significantly downregulated in ALD (*P* < 0.05), which is consistent with the result of transcriptome sequencing (Supplementary Fig. 18).

KEGG pathway analysis indicated that genes related to plant hormone signal transduction, ribosomes, amino acid biosynthesis, starch, and sucrose metabolism, and phenylpropanoid biosynthesis were enriched among the differentially expressed genes between ALF and ALD tissues in Chinese wild rice (Fig. 4e). To elucidate the role of phytohormones in seed shattering in Chinese wild rice, we compared the concentrations of phytohormones between ALF and ALD tissues (Supplementary Table 9). Among them, the concentrations of abscisic acid, ABA-glucosyl ester, 1-aminocyclopropanecarboxylic acid, *cis*-zeatin, *trans*-zeatin riboside, N6-isopentenyladenine, indole-3-acetic acid, 1-*O*-indol-3-ylacetylglucose, indole-3-carboxylic acid, methyl indole-3-acetate, salicylic acid, and salicylic acid 2-*O*-β-glucoside were significantly higher in ALD, whereas those of gibberellin A9, gibberellin A19, jasmonic acid, and methyl jasmonate were significantly lower, than those in ALF (*P* < 0.05) (Supplementary Table 9). Therefore, the concentrations of these phytohormones changed significantly during the process of ALF and ALD in Chinese wild rice.

### Genomic synteny of the phytocassane biosynthetic gene cluster between *O. sativa* and *Z. latifolia*

**Fig. 5:**
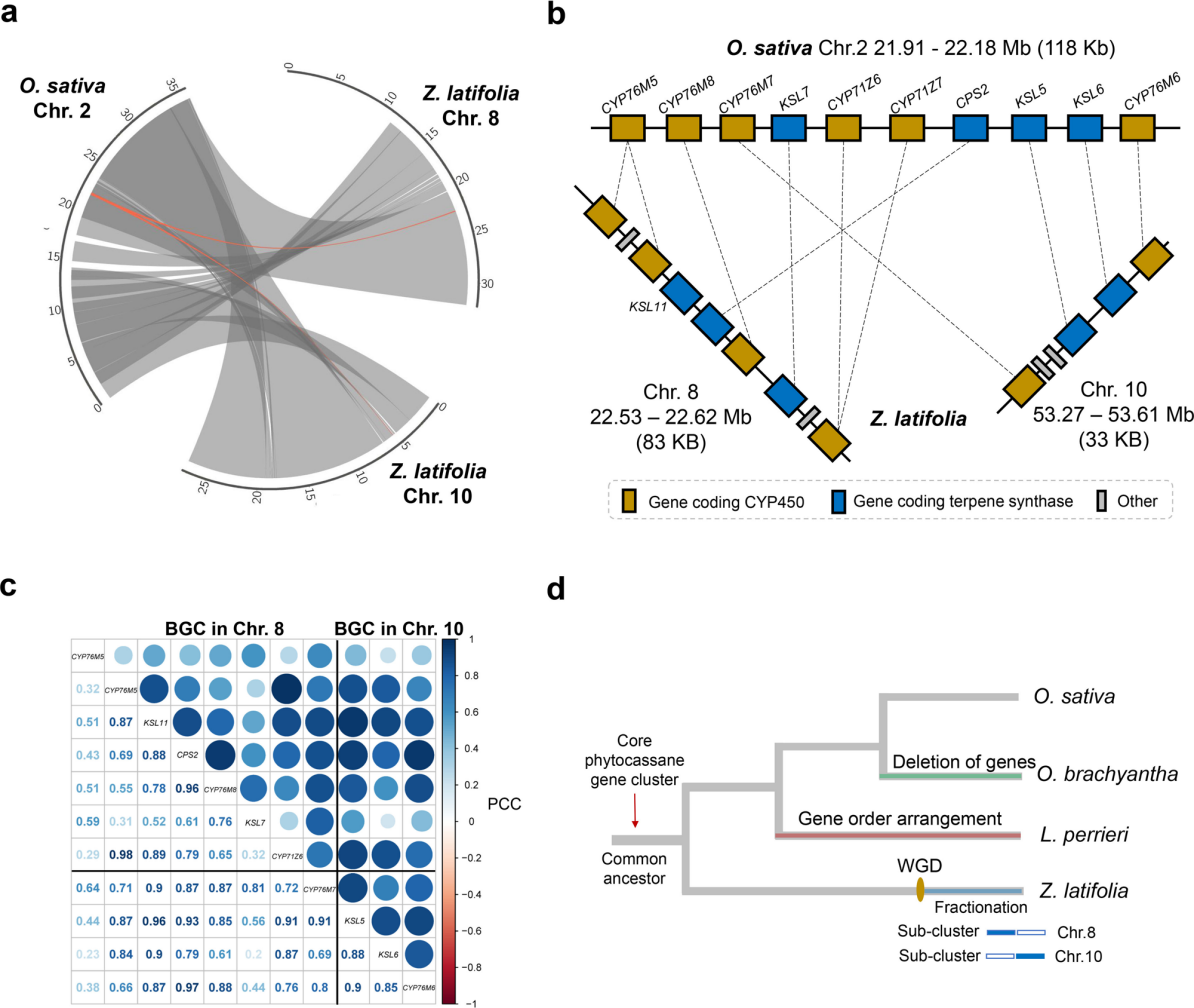
Characterization of the phytocassane biosynthetic gene cluster in *Zizania latifolia* genome. **a**, Genomic synteny between chromosome 2 of *O. sativa* and chromosomes 8 and 10 of *Z. latifolia*. Syntenic genomic blocks are illustrated by the grey lines. The homologous genomic regions of phytocassane biosynthetic gene clusters between *O. sativa* and *Z. latifolia* are highlighted in red. **b** Gene-level synteny between phytocassane biosynthetic gene cluster of *O. sativa* and *Z. latifolia*; CYP450 genes are colored dark yellow; genes coding terpene synthases are colored dark blue. The genes unrelated to the cluster are in grey. **c** Gene co-expression pattern for the genes in the two sub-gene clusters in chromosomes 8 and 10. **d**, Proposed evolutionary history of the phytocassane biosynthetic gene cluster in the *Z. latifolia*, *L. perrieri*, and *Oryza* species.

In our assembled *Z. latifolia* genome, we found genes homologous with *MAS*, *CYP99A*, *CPS*, and *KSL* of the rice momilactone biosynthetic gene cluster. However, the genes most homologous to *MAS*, *CYP99A*, *CPS*, and *KSL* were not located nearby but were scattered among chromosomes 6, 9, 8, and 7, respectively. For the phytocassane biosynthetic gene cluster, we observed two genomic blocks in the *Z. latifolia* genome (Chr. 8: 22.53–22.62 Mb and Chr10: 53.27–53.61 Mb) that showed good synteny with the rice phytocassane biosynthetic gene cluster (Fig. 5a, b). Chromosomes 8 and 10 of *Z. latifolia* were highly colinear with rice chromosome 2, likely owing to a WGD event within the *Zizania* linage after its divergence from *Oryza*. Upon examining the orthologous genes, we found that the candidate clusters on chromosomes 8 and 10 of *Z. latifolia* were not as complete as those in rice but generally complementary to each other (Fig. 5b, Supplementary Table 10). For each sub-cluster of genes on chromosomes 8 and 10, good collinearity was observed with those in rice, despite some rearrangement of the gene order (e.g., *CPS*) (Fig. 5b). Moreover, genes in different sub-clusters showed a highly positive co-expression pattern (Fig. 5c). This suggests that although they are separated into two chromosomes, they are still co-regulated and together play a role in the biosynthesis of phytoalexins.

## Discussion

*Zizania latifolia* is an important aquatic vegetable in East and Southeast Asia, with a high nutritional and medicinal value. Here, we generated a quality-improved genome for Chinese wild rice Huai’an and anchored 99.63% of the sequences to 17 pseudo-chromosomes. Furthermore, our assembly of Huai’an (contig N50 = 4.78 Mb) exhibits a 367.69 ×  longer contig N50 than HSD2 (contig N50 = 13 kb)^31^. Recently, Haas et al.^36^ generated a high-quality genome for northern wild rice and anchored 98.53% of the sequences to 15 chromosomes. The chromosome-level genome will support future studies of molecular genetic breeding and genome evolution in wild rice and the genus *Zizania*.

As an adaptation to the natural environment and offspring propagation, losing seed shattering in rice has been a prime target during plant selection and domestication^21,42^. Recently, Yu et al.^22^ successfully domesticated wild allotetraploid rice (*Oryza alta*) de novo by optimizing the genetic transformation system, assembling the wild allotetraploid rice genome de novo, and editing several genes that control key domestication-related and agronomical traits, such as seed shattering. To the best of our knowledge, *qSH1*, *OsSh1*, and *sh4/SHA1* are the major genes related to rice seed shattering^21^. In Chinese wild rice, three pairs of genes (*ZlqSH1a* and *ZlqSH1b*, *ZlSh1a* and *ZlSh1b*, and *Zlsh4/SHA1a* and *Zlsh4/SHA1b*) showed collinearity with *qSH1*, *OsSh1*, and *sh4/SHA1* in rice but showed a differentially expressed pattern between ALD and ALF. Therefore, the genes related to seed shattering in Chinese wild rice provide a target for reducing its seed shattering and de novo domestication. Notably, genome editing using clustered regularly interspaced short palindromic repeats (CRISPR)/CRISPR-associated protein 9 (Cas9) facilitates targeted genetic manipulation of wild crops and can accelerate crop domestication^43^. Furthermore, the gene editing of major seed-shattering genes by the CRISPR/Cas9 system can aid in development of Chinese wild rice materials with reduced seed-shattering.

An evolutionary history of the two biosynthetic gene clusters in rice was proposed by Miyamoto et al.^44^, who compared genes in the momilactone and phytocassane biosynthetic gene clusters for different *Oryza* species. We found the momilactone biosynthetic gene cluster in the *Z. latifolia* genome, which agrees with the hypothesis of Miyamoto et al.^44^, and this gene cluster evolved within the *Oryza* clade and before the divergence of the rice AA and BB genomes. As for the origin of the phytocassane biosynthetic gene cluster, it is posited that the cluster was present in the common ancestor of the *Oryza* and *Leersia* lineages, with some gene order rearrangements in *L. perrieri*, and gene deletions in some *Oryza* species (Fig. 5d). Based on our findings, the existence of the cluster in the *Z. latifolia* genome suggests that the core phytocassane biosynthetic gene cluster was available in the common ancestor of *Oryza* and *Zizania* species. The two sub-clusters in different chromosomes are likely the result of a recent WGD event within *Zizania* after its divergence from *Oryza*. Additionally, the complementary pattern of the two sub-clusters might be owing to a fractionation process after WGD. Overall, our results provide insights into the genomic evolution process of the phytocassane biosynthetic gene cluster.

In summary, our well-assembled *Z. latifolia* genome will support future basic research on and agronomic improvement of *Z. latifolia* as well as comparative genomic studies between the Gramineae family and other plant species.

## Methods

### DNA extraction and sequencing

The sampling site is located in Baimahu Village, Jinhu County, Huai’an City, Jiangsu Province (33°11′9″ N; 119°9′37″ E)^2^. Owing to the relatively closed geographical environment, Chinese wild rice in this region is highly homozygous. Leaf samples from Chinese wild rice Huai’an were collected for sequencing. After DNA sequencing, reads with adapters, low-quality reads, and reads of <2000 nt were filtered. To determine whether the sequencing data were contaminated, we randomly selected 2,000 single end reads and compared them with those in the Nucleotide Sequence Database by BLAST; there were no contaminated sequences. For Illumina sequencing, a paired-end library with an insert size of 350 bp was sequenced using the Illumina HiSeq X Ten platform (Illumina, San Diego, CA, USA) with a 150 nt layout, according to the manufacturer instructions.

### Genome assembly

Nanopore third-generation sequencing data were corrected using Canu^45^; the SMARTdenovo software (https://hpc.ilri.cgiar.org/smartdenovo-software) was used to assemble the corrected data. The Racon^46^ (https://bioinformaticshome.com/tools/wga/descriptions/Racon.html) and Pilon^47^ software were used to perform three rounds of correction of the third-generation sequencing data and second-generation data, respectively.

The Burrows–Wheeler Alignment software^32^ was used to compare the short sequences obtained from Illumina sequencing with the reference genome in this study, and the integrity of the assembled genome was evaluated through statistical comparisons. The CEGMA v2.5^33^ database containing 458 conserved eukaryotic core genes was used to evaluate the integrity of the final genome assembly. The embryophyta database in OrthoDB v10s (containing 1,614 conserved core genes) and BUSCO v4.0^48^ were used to evaluate the integrity of the genome assembly. Additionally, the LAI value was used to judge the assembly quality based on repetitive genomic regions^34^.

### Hi-C analysis and pseudo-chromosome construction

Fresh young leaves collected from Chinese wild rice Huai’an plants were fixed with 1% formaldehyde. Hi-C fragment libraries were constructed using 300- to 700-bp inserts^49^. The low-quality reads and adapter sequences of raw reads were removed to obtain clean data. Notably, only uniquely aligned paired reads with a mapping quality of >20 were used for further analysis. Before chromosome assembly, we performed a preassembly for error correction of scaffolds, which required the splitting of scaffolds into 50-kb segments. Hi-C data were then mapped to these segments using the Burrows–Wheeler Alignment software. The uniquely mapped data were retained to perform assembly using LACHESIS^50^.

### Repetitive sequence and gene annotation

Using the LTR_FINDER^51^ and RepeatScout^52^ software, we constructed a genome repetitive-sequence database based on ab initio prediction and structure prediction. The database was classified with PASTEClassifier^53^ and then combined with the Repbase database^54^ as the final repeat-sequence database. We then used RepeatMasker^55^ for repetitive-sequence prediction of the genome. Default parameters were used for LTR_FINDER, RepeatScout, and PASTEClassifier, and the ‘-nolow -no_is -norna -engine wublast’ parameter was used for RepeatMasker. Additionally, we used the EDTA (v1.9.7) software to generate TE annotations^56^.

The gene-structure prediction was performed for the *Z. latifolia* genome using ab initio prediction, prediction based on homologous species, and prediction based on Unigene analysis. The prediction results were integrated using EVM v1.1.1^57^. First, we used Genscan^58^, Augustus v2.4^59^, GlimmerHMM v3.0.4^60^, GeneID v1.4^61^, and SNAP^62^ for ab initio prediction. Second, we used GeMoMa v1.3.1^63,64^ for prediction based on homologous species. Stringtie v1.2.3^65^ and Hisat v2.0.4^66^, and GeneMarkS-T v5.1^67^ and TransDecoder v2.0, were used for assembly and gene prediction, respectively. We sequenced the mixed RNA library generated from the root, stem, leaf, leaf sheath, male and female florets, seed, and a whole un-emerged panicle for transcriptome-based predictions. Additionally, the RNA-seq reads were assembled into transcripts using Trinity v2.1.1^68^, and PASA v2.0.2^69^ was used to predict the Unigene based on RNA-seq reads.

Noncoding RNAs include miRNA, rRNA, tRNA, and other RNAs with known functions. Blastn was used for genome-wide alignment based on the Rfam database^70^ to identify miRNAs and rRNAs, and tRNAscan-SE^71^ was used to identify tRNAs. The predicted protein sequences were used to search for homologous gene sequences through GenBlastA^72^ alignment, and GeneWise^73^ was then used to search for premature stop codons and frameshift mutations that resulted in pseudogenes. For GenBlastA, an e-value of 1 × 10^−5^ was used; all other parameters were set to default. Additionally, default parameters were used for GeneWise. BLAST v2.2.31^74^ alignment (e-value: 1 × 10^−5^) was performed between the predicted gene sequence and the Non-Redundant Protein Sequence Database^75^, EuKaryotic Orthologous Groups^76^, Gene Ontology^77^, KEGG^78^, and TrEMBL^79^ functional databases.

### Gene families and phylogenetic analysis

Orthofinder v2.4^80^ was used to classify the protein sequences of nine gramineous plants (*B. distachyon*, *H. vulgare*, *L. perrieri*, *O. brachyantha*, *O. sativa*, *S. bicolor*, *S. italica*, *Z. latifolia*, and Z*. mays*) and one dicotyledon (*A. thaliana*) into families. The PANTHER V15 database^81^ was used for annotation of the gene families obtained. IQ-TREE v1.6.11^82^ was used to construct a phylogenetic tree from 1,371 single-copy protein sequences. Specifically, MAFFT v7.205 (https://mafft.cbrc.jp/alignment/software/) was used to align each single-copy gene family sequence, and the PAL2NAL v14 program^83^ was then used to convert the protein alignment to codon alignment. We then used Gblocks v0.91b (parameter: -b5 = h)^84^ to remove regions with large differences or poor sequence alignment. Finally, the aligned gene family sequences of each species were connected end-to-end to obtain a super-gene alignment. The model testing tool ModelFinder^85^, included with IQ-TREE (http://www.iqtree.org/), was used for model selection, with the best model identified as GTR + F + I + G4. Using this model, we applied the maximum-likelihood method to construct a phylogenetic tree, with the number of bootstraps set to 1,000. The outgroup of the obtained phylogenetic tree was set as *A. thaliana*, which gave a rooted tree, and the MCMCTREE package included in the PAML v4.9i software^86^ was then used to calculate divergence times. The final phylogenetic tree with divergence times was displayed graphically using MCMCTreeR v1.1^87^. CAFE v4.2^88^ was used with the phylogenetic tree with divergence times and genes (after clustering into families) to estimate the number of gene family members of an ancestor from each branch through birth-death models, predicting the contraction and expansion of a gene family from each species relative to that of its ancestor. Significant expansion or contraction was defined as family-wide *p* values and viterbi *p* values (both < 0.05).

We used the CodeML module in PAML for positive-selection analysis. First, we obtained single-copy gene families common among *B. distachyon*, *H. vulgare*, *L. perrieri*, *O. brachyantha*, *O. sativa*, and *Z. latifolia*, followed by MAFFT (parameters: —localpair —maxiterate 1000) alignment of the protein sequences of each gene family and conversion to the codon alignment sequence using PAL2NAL. Finally, CodeML was used to perform likelihood ratio tests of model A and the null model using the ‘chi2’ program in PAML based on the branch-site model. An empirical Bayes method was used to obtain the posterior probability of being considered a positively selected site (>0.95 is usually considered a significantly positively selected site).

### Collinearity and WGD analyses

We used Diamond v0.9.29.130^89^ to compare the protein sequences of *O. sativa* and *Z. latifolia* (C-score > 0.5; *e* value < 1 × 10^−5^). Subsequently, we identified the collinear blocks between the genomes of *O. sativa* and *Z. latifolia* using MCScanX^90^. Finally, based on the distribution of the Ks paralogous genes, we calculated the WGD events using the WGD software^91^.

### Identification of seed-shattering genes in Chinese wild rice

Genes related to seed shattering in *O. sativa* were obtained by querying the gene name on the website of the China Rice Data Centre (https://www.ricedata.cn/). Seed-shattering genes in *Z. latifolia* were obtained by comparing similar genes in *O. sativa* with the genome sequences of *Z. latifolia* in this study. The e-value of the sequence-alignment results was set to <1 × 10^−10^. MCScanX was used for collinearity analysis of candidate genes.

### Histologic analysis of the anatomic structure of the abscission layer

The ALF and ALD tissues (1–2 mm above and below, respectively, the junction between the flower and the pedicel) were collected, and a freehand longitudinal section was prepared using a thin blade. The collected sample was stained using a 0.1% aqueous solution of Acridine Orange for 10–15 min, rinsed three times with deionized water, placed on a glass slide, and observed under a confocal laser microscope (Leica SP8; Leica Biosystems, Nussloch, Germany) at 488 and 543 nm.

### Transcriptome analysis

Transcriptome sequencing analysis of the ALF and ALD tissues was performed according to the method of Yan et al.^92^. Gene-expression levels were quantified by estimating fragments per kilobase of transcript per million fragments mapped. The genes with an adjusted *P* < 0.01 according to DESeq2 were identified as differentially expressed. We used KOBAS^93^ to test the enrichment of differentially expressed genes in the KEGG pathways.

### Data validation by real-time PCR

The qRT-PCR analysis was performed on eight selected seed-shattering genes between ALF and ALD. The method of qRT-PCR was performed according to Wang et al^9^. The primers used for detecting the expression levels of the genes are listed in Supplementary Table 11.

### Biosynthetic gene clusters between *O. sativa* and *Z. latifolia*

The protein sequences encoded by the genes in two known rice biosynthetic gene clusters were identified^26,27^ and used to search for orthologous genes in *Z. latifolia*. Syntenic genomic blocks between chromosome 2 of *O. sativa* and chromosomes 8 and 10 of *Z. latifolia* were identified using the MCScan program^90^ and visualized by Circos^94^. Based on 12 transcriptomes from tissues of ALF, ALD, leaf, and stem, the expression levels of genes related to the phytocassane biosynthetic gene cluster were extracted, and the co-expression coefficient matrix was visualized using the R package ‘corrplot’^95^.

### Detection of phytohormones

Fresh plant materials (ALF and ALD tissues) were harvested, weighed, immediately frozen in liquid nitrogen, and stored at −80 °C until needed. Plant materials (50 mg fresh weight) were frozen in liquid nitrogen, ground to powder, and extracted with 1 mL of methanol/water/formic acid (15:4:1, v/v/v). Phytohormone contents were detected using MetWare (http://www.metware.cn/) based on the AB Sciex QTRAP 6500 LC-MS/MS platform.

### Statistics and reproducibility

In Supplementary Fig. 18 and Supplementary Table 9, we used *n* = 3 biologically independent samples. Statistical significance was assessed using a two-tailed Student’s *t*-test, *P < 0.05.

### Reporting summary

Further information on research design is available in the Nature Research Reporting Summary linked to this article.

## Supplementary information

Supplementary Information

Description of Additional Supplementary Files

Supplementary Data 1

Supplementary Data 2

Supplementary Data 3

Supplementary Data 4

Reporting Summary

## Data Availability

Raw reads and transcriptome sequencing data have been deposited in GenBank under the accession number PRJNA719466. The whole genome sequence data have been deposited in Genome Sequence Archive under the accession number GWHBFHI00000000, which is publicly accessible at https://ngdc.cncb.ac.cn/gwh.
