# Supplementary material for: Chromosome-level genome assembly of Zizania latifolia provides insights into its seed shattering and phytocassane biosynthesis
Source: Commun Biol. 2022 Jan 11;5:36. doi: 10.1038/s42003-021-02993-3 (PMC8752815; doi:10.1038/s42003-021-02993-3)
Supplement: Supplementary file 1 — Supplementary Information [file 42003_2021_2993_MOESM1_ESM.pdf]

Supplementary Information for

Chromosome-level genome assembly of *Zizania latifolia* provides insights into its seed shattering and phytocassane biosynthesis

Ning Yan [1,#✉], Ting Yang [1,#], Xiu-Ting Yu [1,2,#], Lian-Guang Shang [3,#], De-Ping Guo [4], Yu Zhang [1], Lin Meng [1], Qian-Qian Qi [1,2], Ya-Li Li [1,2], Yong-Mei Du [1], Xin-Min Liu [1], Xiao-Long Yuan [1], Peng Qin [5], Jie Qiu [6], Qian Qian [7✉], Zhong-Feng Zhang [1✉]

✉Correspondence should be addressed to N.Y. (yanning@caas.cn), Q.Q. (qianqian188@hotmail.com), or Z.-F.Z. (zhangzhongfeng@caas.cn).

[1] Tobacco Research Institute of Chinese Academy of Agricultural Sciences, Qingdao 266101, China

[2] Graduate School of Chinese Academy of Agricultural Sciences, Beijing 100081, China

[3] Shenzhen Branch, Guangdong Laboratory of Lingnan Modern Agriculture, Genome Analysis Laboratory of the Ministry of Agriculture and Rural Affairs, Agricultural Genomics Institute at Shenzhen, Chinese Academy of Agricultural Sciences, Shenzhen 518120, China

[4] Department of Horticulture, College of Agriculture and Biotechnology, Zhejiang University, Hangzhou 310058, China

[5] State Key Laboratory of Crop Gene Exploration and Utilization in Southwest China, Rice Research Institute, Sichuan Agricultural University, Chengdu, Sichuan 611130, China

[6] Shanghai Key Laboratory of Plant Molecular Sciences, College of Life Sciences, Shanghai Normal University, Shanghai 200234, China

[7] State Key Laboratory of Rice Biology, China National Rice Research Institute, Chinese Academy of Agricultural Sciences, Hangzhou 310006, China

[#] These authors contributed equally: Ning Yan, Ting Yang, Xiu-Ting Yu, Lian-Guang Shang

This PDF file includes:

Supplementary Figs. 1–18

Supplementary Tables 1–11

Supplementary References

Supplementary Figures

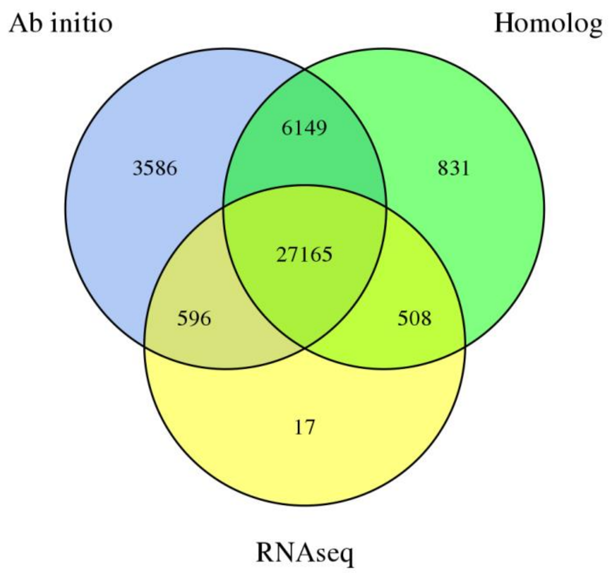

Supplementary Fig. 1. Distribution of Chinese wild rice genes derived from three prediction methods after EVidenceModeler integration.

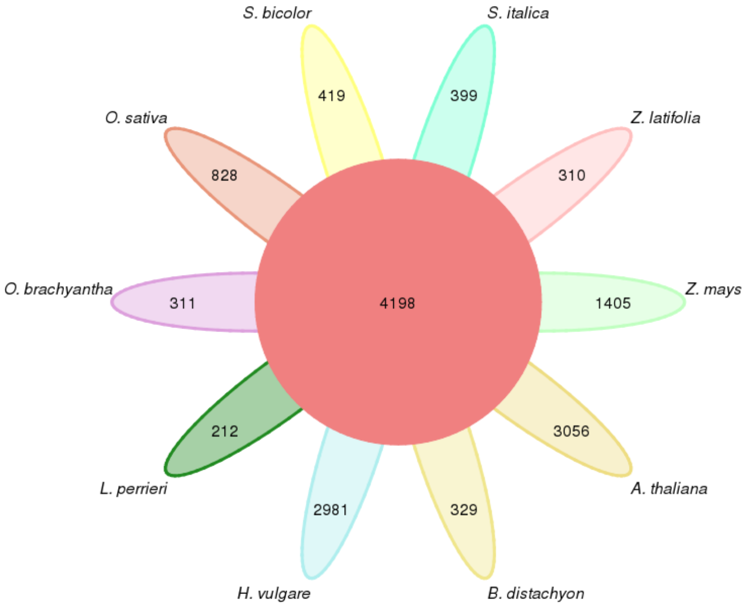

Supplementary Fig. 2. Petal diagram of gene family clustering of *Zizania latifolia* and nine other species. The number of gene families shared by all species is shown in the middle circle and the number of gene families unique to each species is shown in the outer edges.

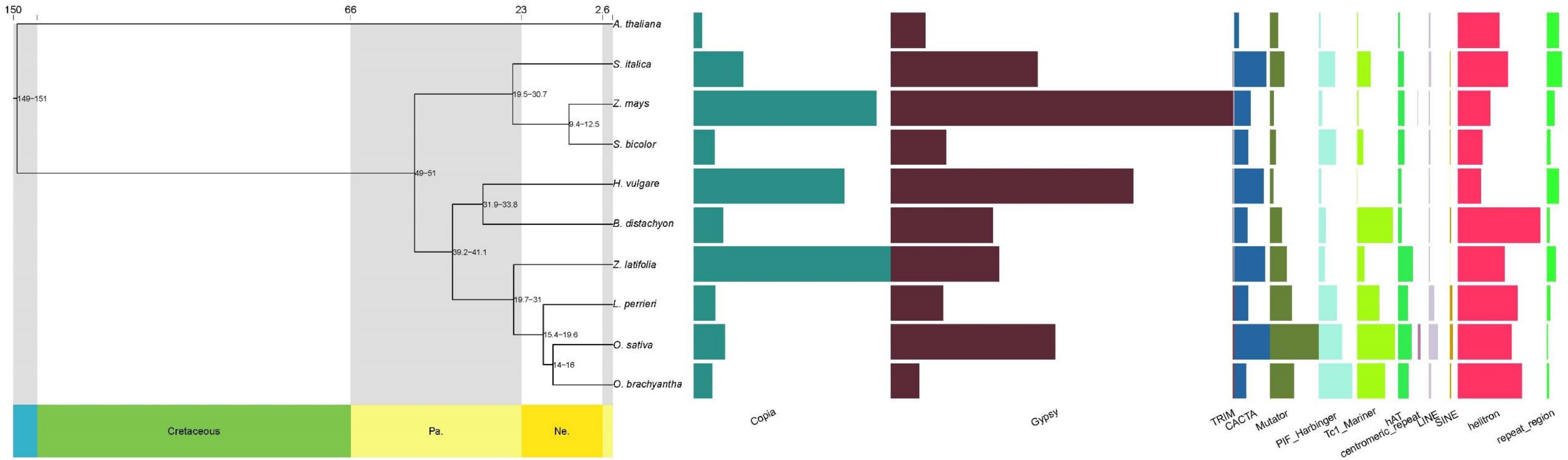

Supplementary Fig. 3. Evolutionary trees and transposable element (TE) of Chinese wild rice and other representative plant species with differentiation time. Time on the evolutionary tree represents the divergence time supported by 95% of the highest posterior density (HPD). At the bottom of the tree is the geological era, and at the top of the tree is the absolute age, in millions of years, defined by the shadow of each geological period. The geological periods include Cretaceous, Paleogene (Pa.), and Neogene (Ne.). Each coloured bar represents the proportion of each TE superfamily.

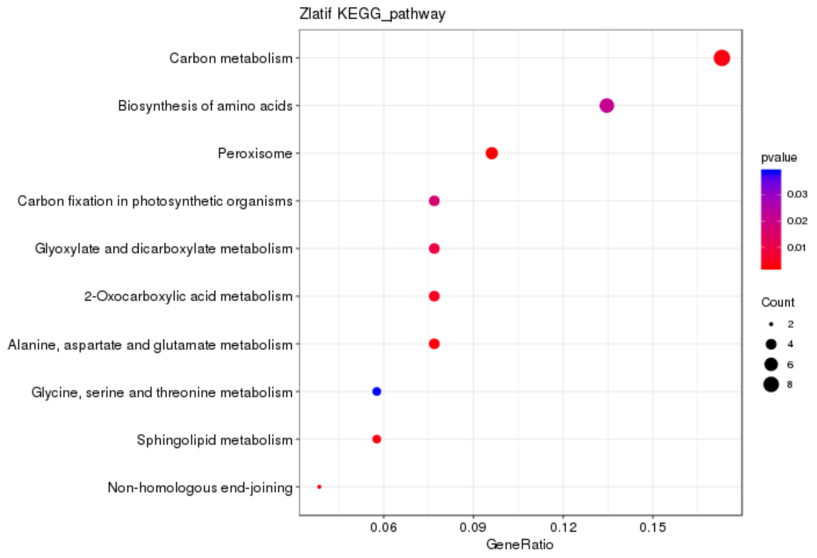

Supplementary Fig. 4. KEGG enrichment analyses for the positive selection genes in *Zizania latifolia*.

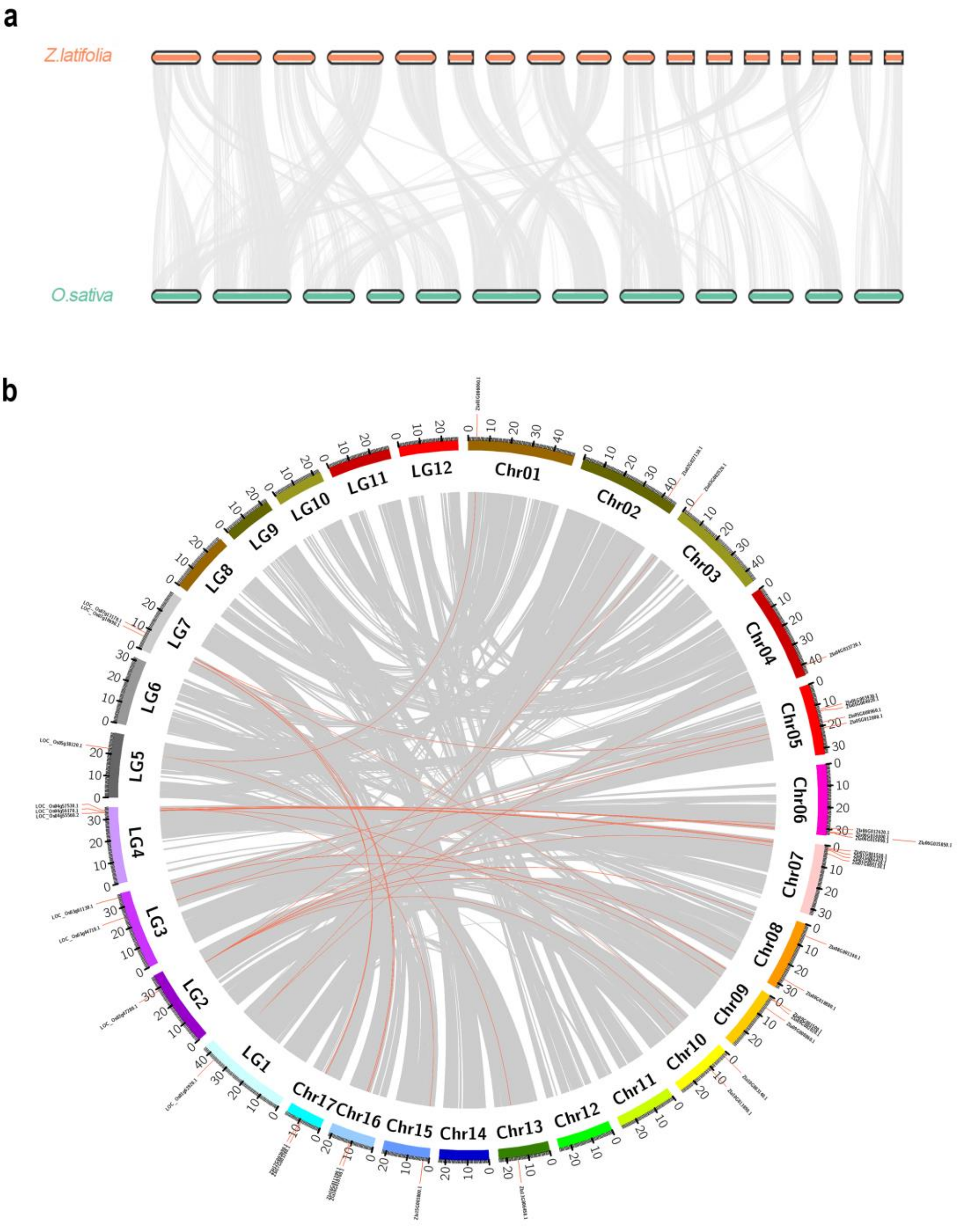

Supplementary Fig. 5. Collinearity analysis between genomes (a) and seed shattering-related genes (b) of *Zizania latifolia* and *Oryza sativa*. Chr01–Chr17, chromosomes 1–17 of *Z. latifolia*; LG1–LG12; linkage groups 1–12 of *O. sativa*. Candidate genes for seed shattering in *O. sativa* and *Z. latifolia* are shown in Supplementary Data 4. The grey lines represent the collinearity between the *O. sativa* and *Z. latifolia* genome, and the red lines represent the collinearity of seed shattering-related genes between *Z. latifolia* and *O. sativa*.

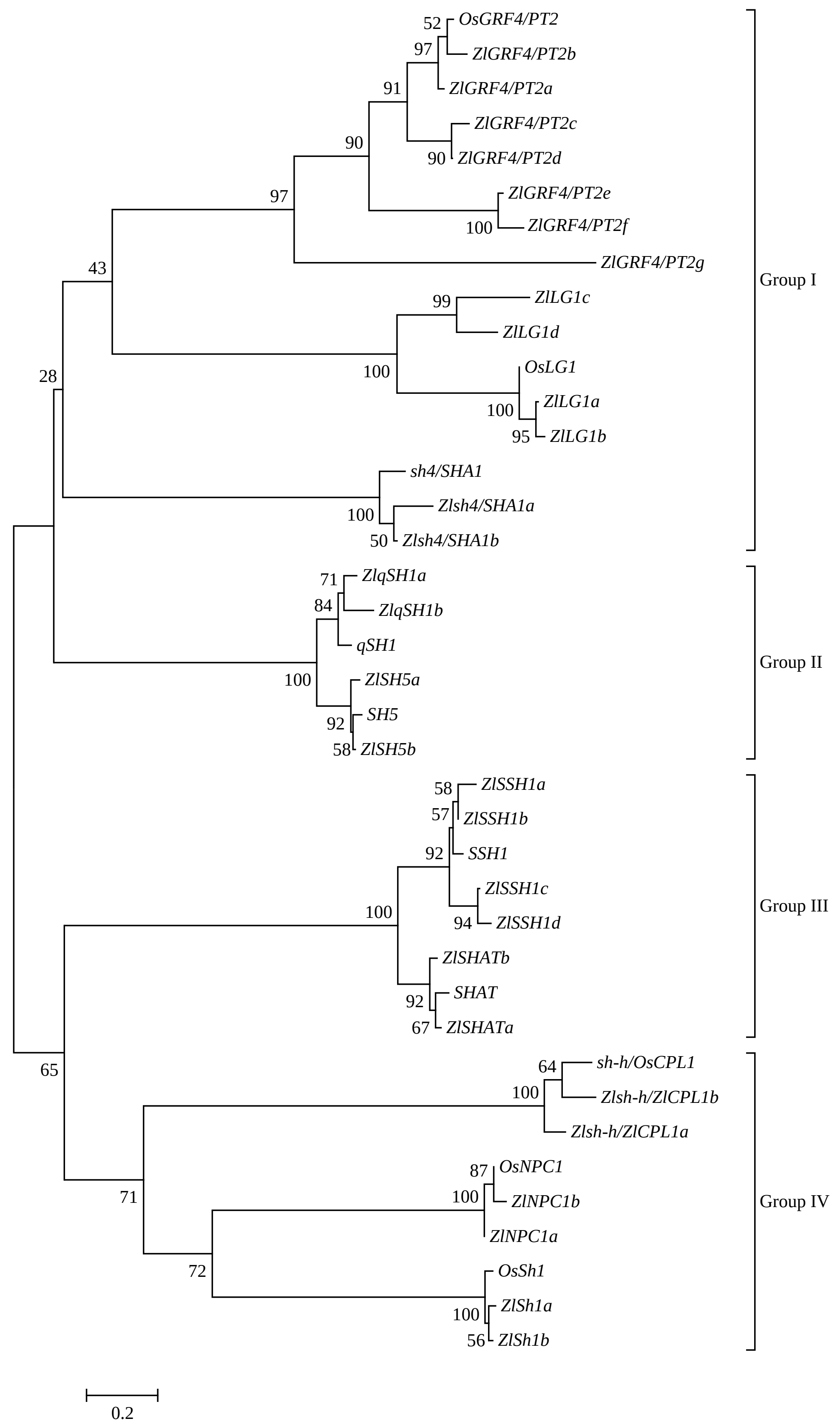

Supplementary Fig. 6. A phylogenetic tree of seed shattering genes in *Zizania latifolia* and *Oryza sativa*.

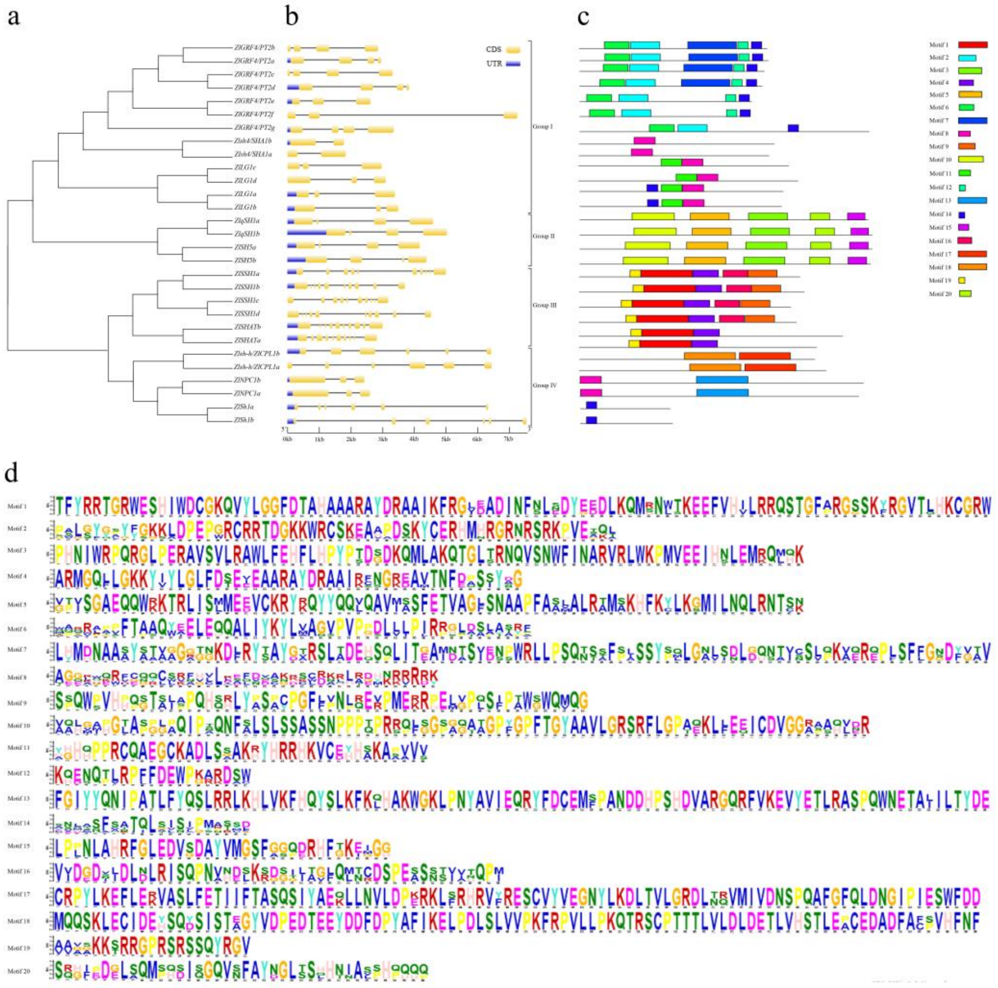

Supplementary Fig. 7. Analysis on motif of seed shattering genes in *Zizania latifolia*. **a,** phylogenetic tree; **b,** gene structure analysis; **c,** motif distribution analysis; **d,** motif sequence analysis.

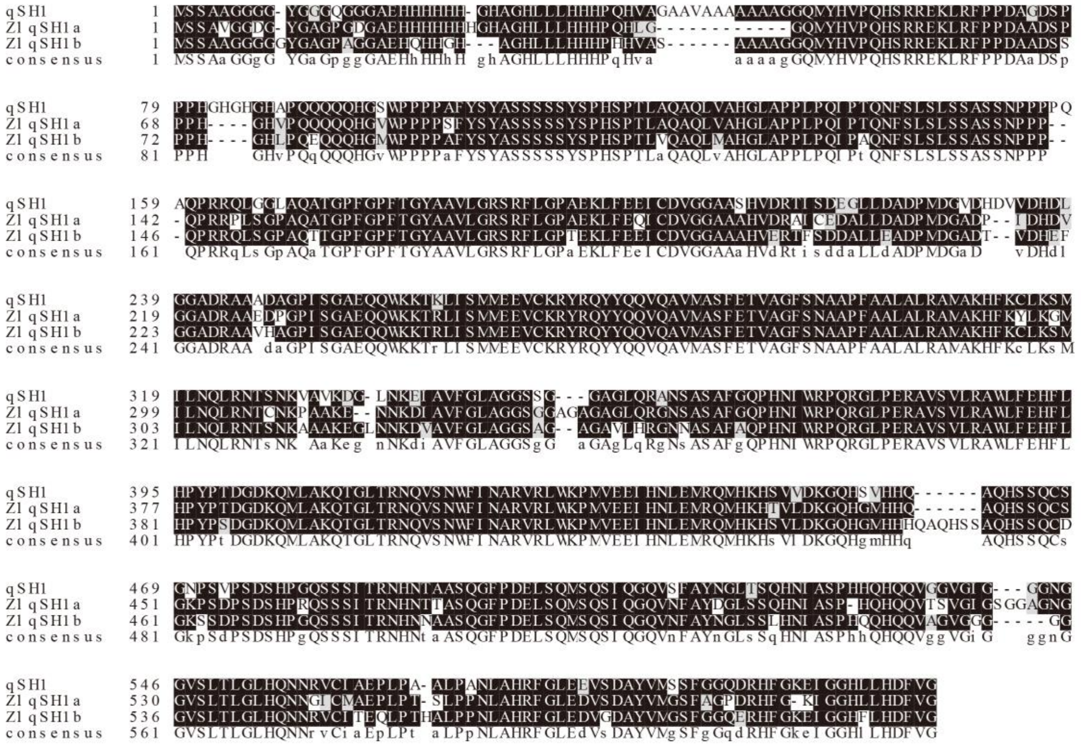

Supplementary Fig. 8. The protein sequence alignment of qSH1 and its homologous sequences in *Z. latifolia* and *Oryza* species.

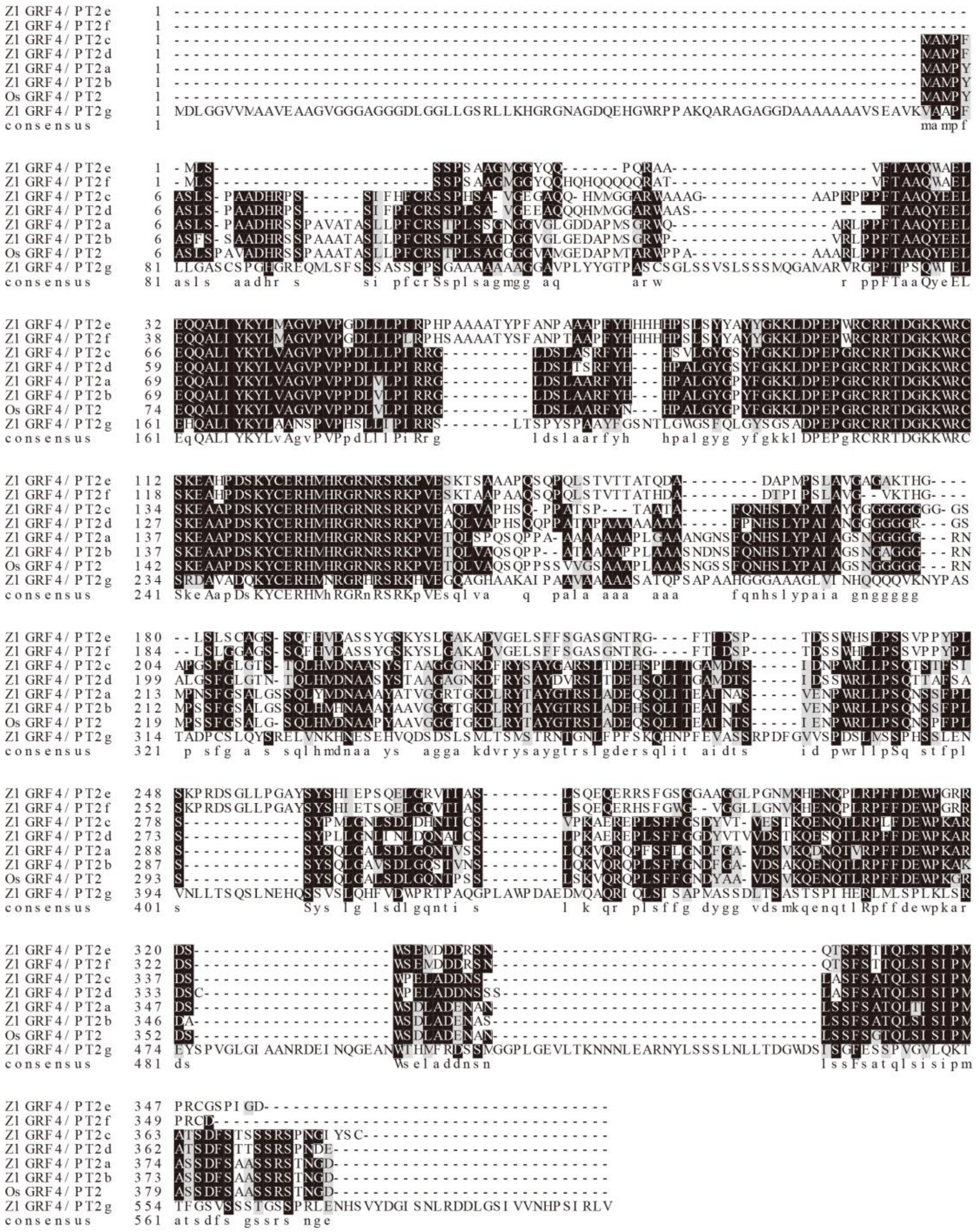

Supplementary Fig. 9. The protein sequence alignment of OsGRF4/PT2 and its homologous sequences in *Z. latifolia* and *Oryza* species.

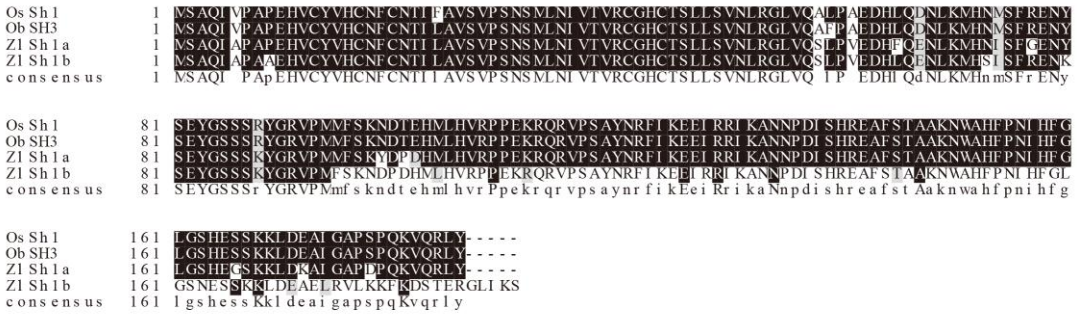

Supplementary Fig. 10. The protein sequence alignment of OsSh1 and its homologous sequences in *Z. latifolia* and *Oryza* species.

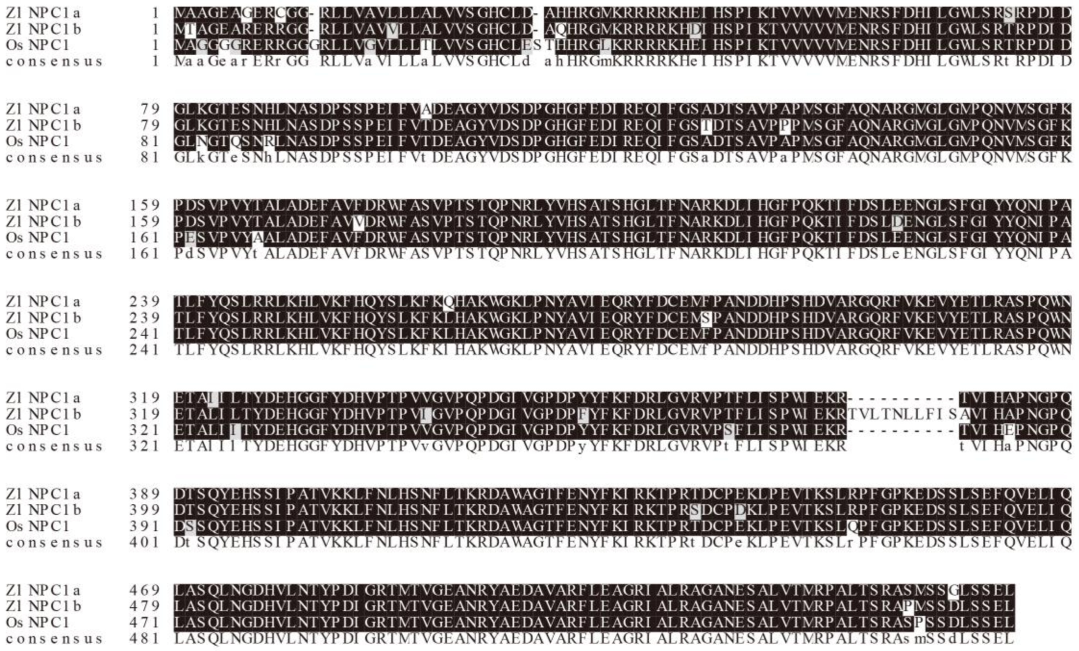

Supplementary Fig. 11. The protein sequence alignment of OsNPC1 and its homologous sequences in *Z. latifolia* and *Oryza* species.

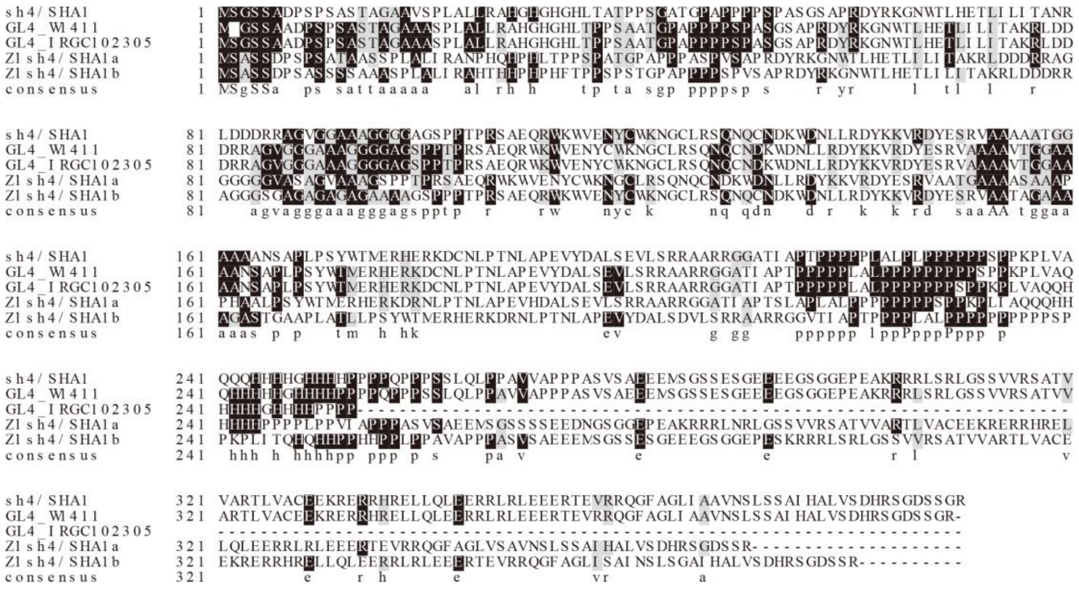

Supplementary Fig. 12. The protein sequence alignment of sh4/SHA1 and its homologous sequences in *Z. latifolia* and *Oryza* species.

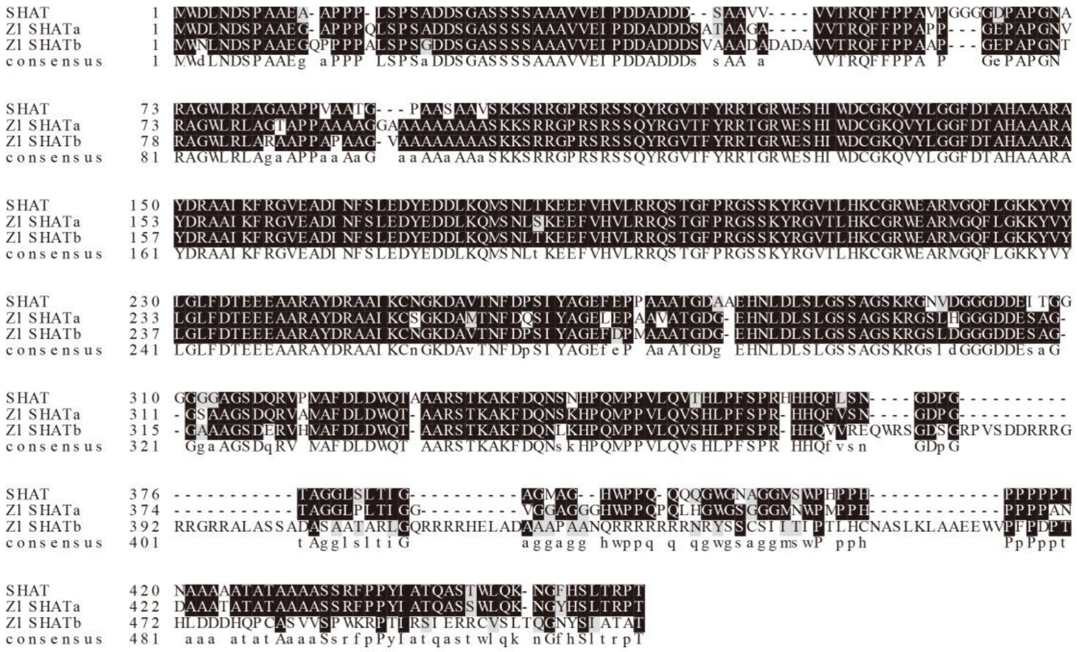

Supplementary Fig. 13. The protein sequence alignment of SHAT1 and its homologous sequences in *Z. latifolia* and *Oryza* species.

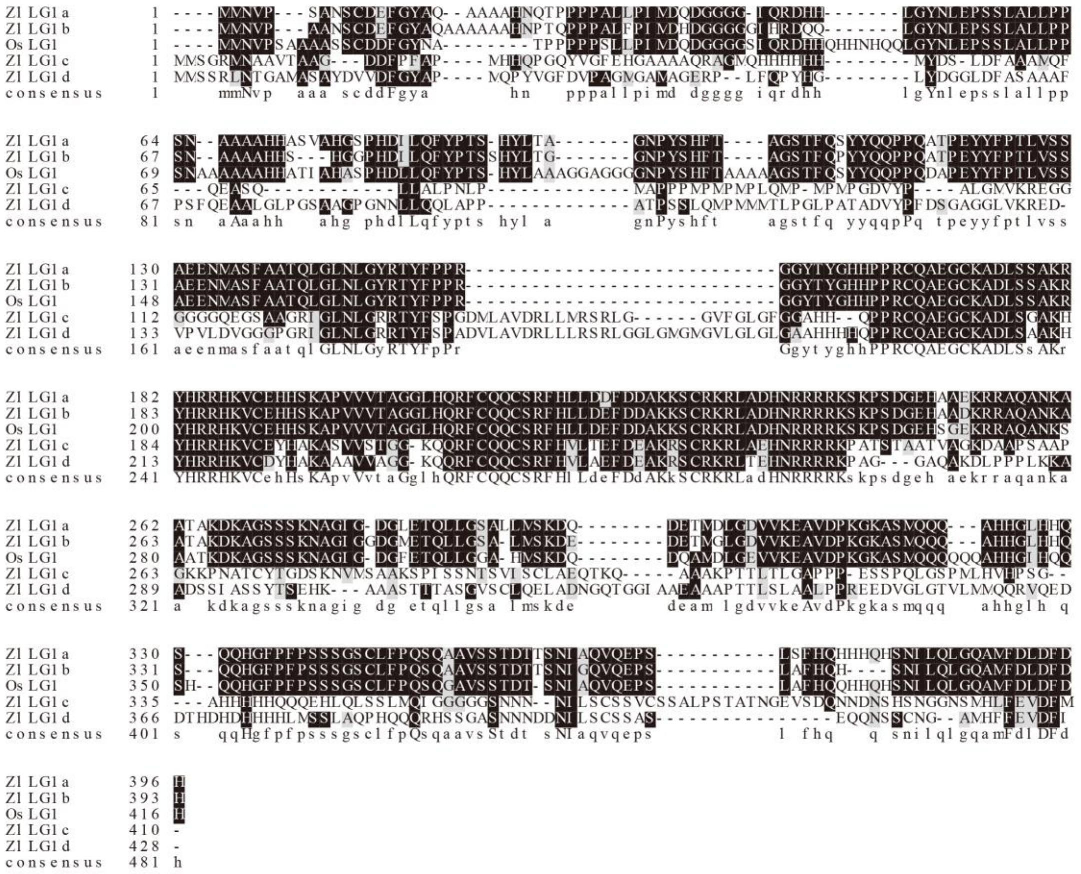

Supplementary Fig. 14. The protein sequence alignment of OsLG1 and its homologous sequences in *Z. latifolia* and *Oryza* species.

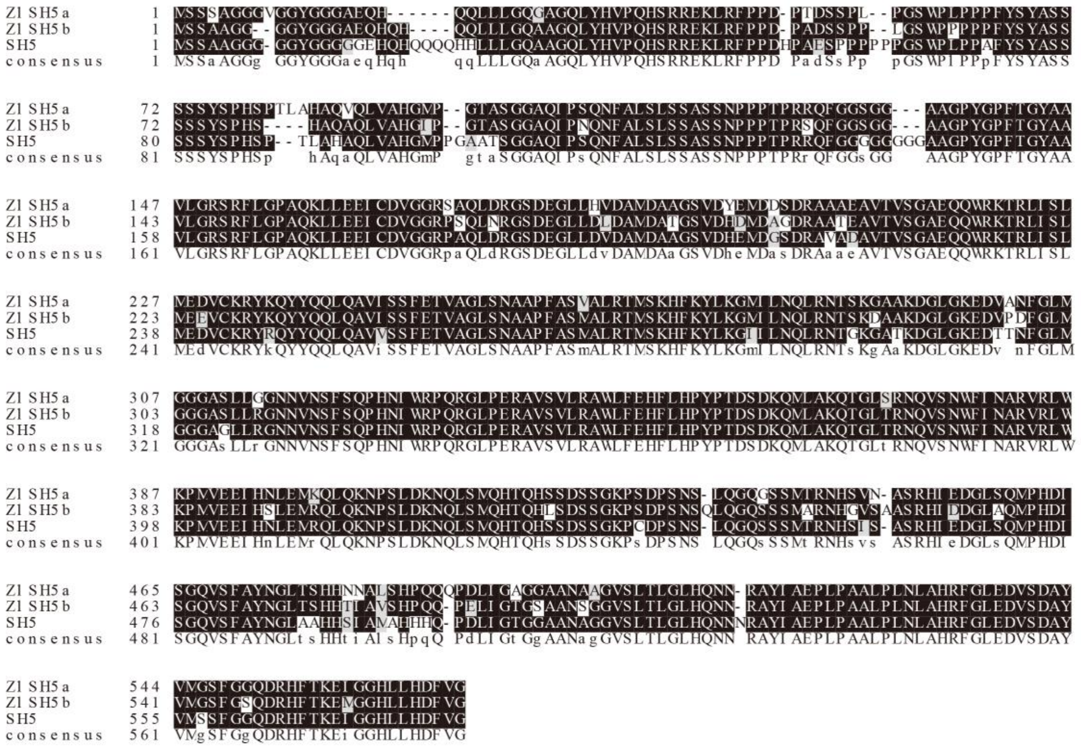

Supplementary Fig. 15. The protein sequence alignment of SH5 and its homologous sequences in *Z. latifolia* and *Oryza* species.

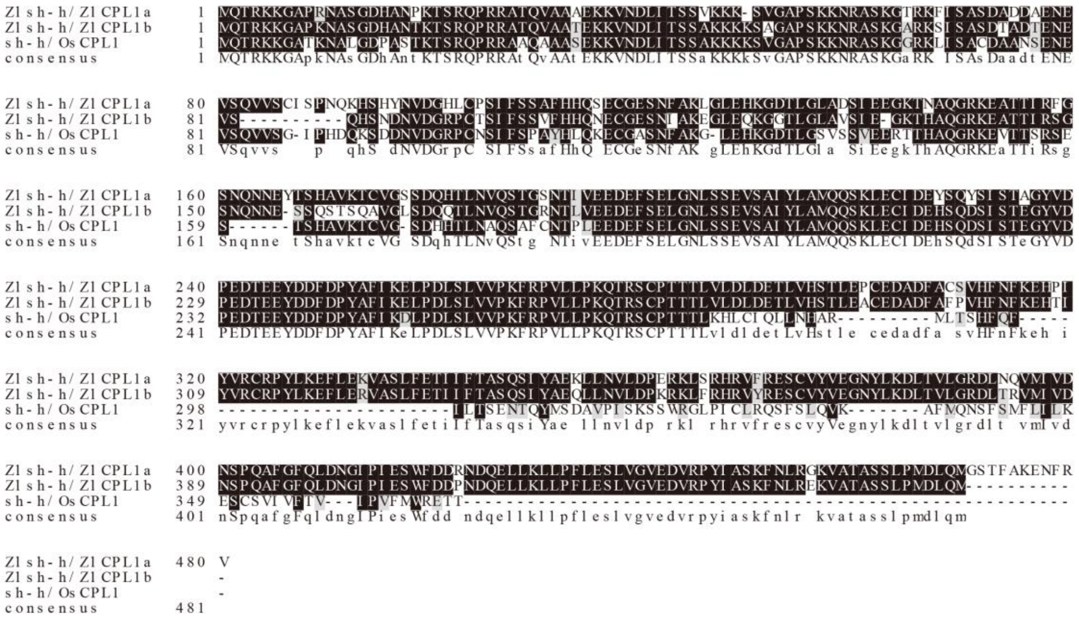

Supplementary Fig. 16. The protein sequence alignment of sh-h/OsCPL1 and its homologous sequences in *Z. latifolia* and *Oryza* species.

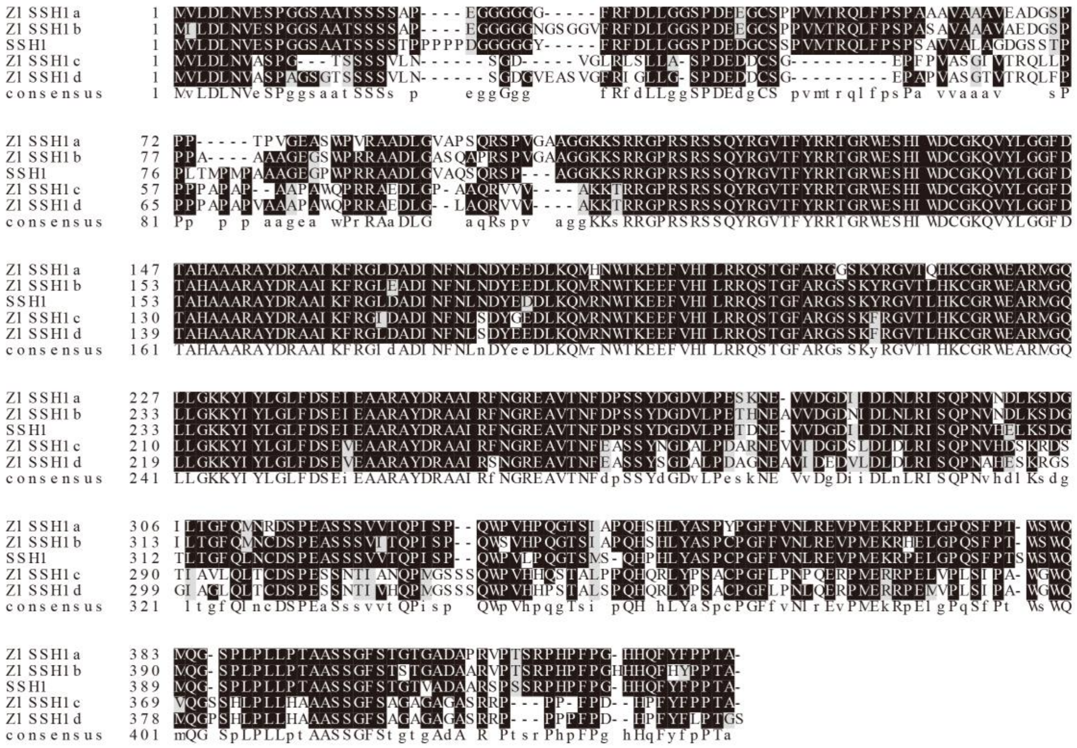

Supplementary Fig. 17. The protein sequence alignment of SSH1 and its homologous sequences in *Z. latifolia* and *Oryza* species.

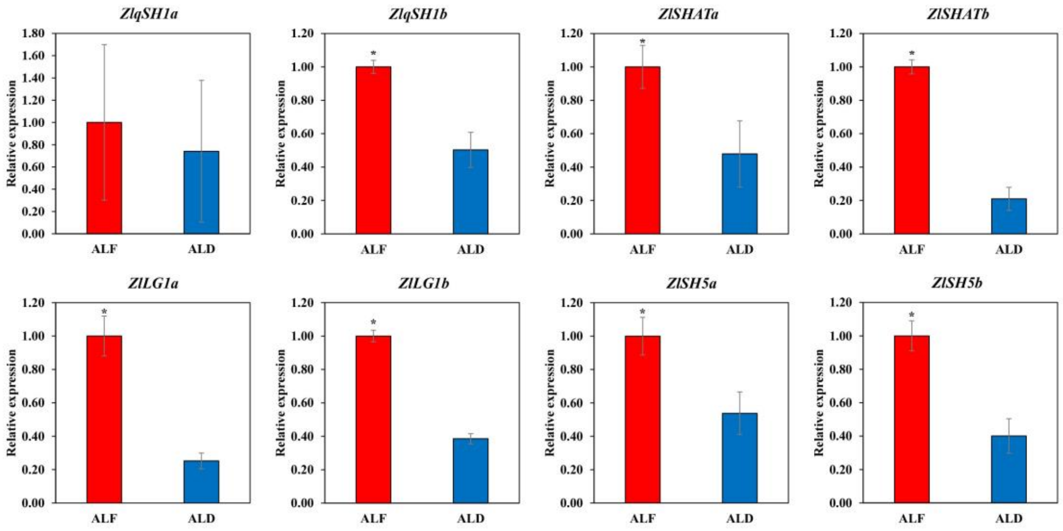

Supplementary Fig. 18. Gene expression analyses of the selected seed shattering genes in *Zizania latifolia* as measured by qRT-PCR. Error bars indicate the standard error of the mean of the three independent experiments. Asterisks (*) indicate values that differ significantly according to Tukey's test ($P < 0.05$).

Supplementary Tables

Supplementary Table 1. Sequencing data statistics of Oxford Nanopore technology (ONT) and Illumina sequencing for Chinese wild rice Huai'an

**Clean Reads**	**ONT sequencing**		**Illumina sequencing**
	**Raw reads**	**Clean reads**	**Clean reads**
Size of library (bp)	20	20	350
Number of reads	5,009,027	3,970,614	228,677,035
Average length of reads (bp)	13,067	15,503	150
Total bases (bp)	65,456,644,980	61,559,056,417	68,499,340,900
Mean quality	7.89	8.37	Q30 > 92.19%
Sequencing depth	119.58×	112.46×	113.01×

Supplementary Table 2. Statistics of 17 Chinese wild rice assembled chromosomes

Group	Cluster number	Cluster length (bp)	Order number	Order length (bp)
Chr01	18	50,101,234	13	49,612,983
Chr02	29	48,046,012	19	47,112,129
Chr03	21	46,583,488	10	45,588,614
Chr04	12	45,369,969	8	44,977,458
Chr05	18	33,710,764	10	32,992,499
Chr06	12	33,104,032	9	32,889,764
Chr07	21	33,547,554	15	32,783,925
Chr08	12	32,655,998	8	32,287,380
Chr09	17	28,790,088	9	28,249,098
Chr10	25	29,018,640	14	27,421,652
Chr11	11	27,637,196	7	27,423,664
Chr12	22	26,660,827	14	25,649,991
Chr13	12	23,914,037	7	23,650,067
Chr14	31	24,459,573	21	23,072,643
Chr15	10	22,050,986	4	21,753,664
Chr16	16	22,318,559	10	21,397,443
Chr17	13	17,392,854	7	17,007,237
Total (ratio %)	300(90.36)	545,361,811(99.63)	185(61.67)	533,870,211(97.89)

Supplementary Table 3. Evaluation of genome assembly completeness with 1,375 BUSCO[1] groups

	Genome	
**Type**	**Number**	**Percent (%)**
Complete BUSCOs	1,577	97.71
Complete and single-copy BUSCOs	1249	77.39
Complete and duplicated BUSCOs	328	20.32
Fragmented BUSCOs	9	0.56
Missing BUSCOs	28	1.73
Total Lineage BUSCOs	1,614	100

[1]BUSCO, Benchmarking Universal Single-Copy Orthologue

Supplementary Table 4. Comparison of BUSCO groups of Next Generation Sequencing (NGS) and Oxford Nanopore technology (ONT) of the Chinese wild rice genomes

Type	Complete BUSCOs[1]	Complete and single-copy BUSCOs	Complete and duplicated BUSCOs	Fragmented BUSCOs	Missing BUSCOs	Total lineage BUSCOs
NGS	4426 (90.40%)	3208 (65.52%)	1218 (24.88%)	96 (1.96%)	374 (7.64%)	4896
ONT	4634 (94.65%)	3130 (63.93%)	1504 (30.72%)	37 (0.76%)	225 (4.60%)	4896

[1]BUSCO, Benchmarking Universal Single-Copy Orthologue

Supplementary Table 5. Comparison of long terminal repeat (LTR) assembly index (LAI) of Next Generation Sequencing (NGS) and Oxford Nanopore technology (ONT) of the Chinese wild rice genome

Type	From	To	Intact	Total	Raw LAI	LAI
NGS	1	603,989,347	0.0022	0.1782	1.25	6.88
ONT	1	547,397,560	0.0299	0.3773	7.94	13.57

Supplementary Table 6. Statistics of repetitive sequences in the Chinese wild rice genome

Type	Number	Length (bp)	Rate (%)
ClassI	244,890	206,264,154	37.68
ClassI/LTR/Copia	120,603	124,698,688	22.78
ClassI/LTR/Gypsy	89,814	68,441,219	12.50
ClassI/LTR/TRIM	2,276	416,227	0.08
ClassI/LTR/Unknown	30,558	12,172,708	2.22
ClassI/LINE	1,354	490,060	0.09
ClassI/SINE	284	44,904	0.01
ClassI/Unknown	1	348	0
ClassII	263,498	83,298,065	15.21
ClassII/TIR/CACTA	41,332	19,593,368	3.58
ClassII/TIR/Mutator	35,375	10,591,002	1.93
ClassII/TIR/PIF_Harbinger	14,615	3,554,936	0.65
ClassII/TIR/Tc1_Mariner	19,714	4,591,368	0.84
ClassII/TIR/hAT	31,088	9,096,346	1.66
ClassII/TIR/polinton	13	6,986	0
ClassII/TIR/Unknown	2,208	857,661	0.16
ClassII/helitron	102,073	29,475,694	5.38
ClassII/repeat_region	17,080	5,530,704	1.01
Total	508,388	289,562,219	52.89

Supplementary Table 7. Statistics of gene family clustering in 11 species

Species	Number of genes	Number of genes in orthogroups	Number of un-assigned genes	Percentage of genes in ortho-groups (%)	Percentage of unassigned genes (%)	Number of orthogroups containing species	Percentage of orthogroups containing species (%)	Number of species-specific orthogroups	Number of genes in species-specific orthogroups	Percentage of genes in species-specific orthogroups (%)
Arabidopsis thaliana	27,379	15,356	12,023	56.1	43.9	9,637	25.2	3,056	7,926	28.9
Brachypodium distachyon	32,439	26,402	6,037	81.4	18.6	20,713	54.3	329	957	3.0
Hordeum vulgare	63,658	50,307	13,351	79.0	21.0	23,472	61.5	2,981	17,345	27.2
Leersia perrieri	29,017	24,065	4,952	82.9	17.1	19,855	52.0	212	586	2.0
Oryza brachyantha	32,010	22,966	9,044	71.7	28.3	19,528	51.2	311	733	2.3
Oryza sativa	42,189	31,919	10,270	75.7	24.3	22,797	59.7	828	3,278	7.8
Sorghum bicolor	34,129	28,190	5,939	82.6	17.4	21,634	56.7	419	1,206	3.5
Setaria italica	34,584	29,831	4,753	86.3	13.7	21,966	57.5	399	1,363	3.9
Zizania latifolia	38,852	33,924	4,928	87.3	12.7	20,342	53.3	310	788	2.0
Zea mays	39,422	33,356	6,066	84.6	15.4	21,092	55.3	1,405	4,661	11.8

Supplementary Table 8. Seed shattering related genes in *Oryza sativa*

Gene	Chromosome	Genomic locus	Coding protein	Reference
qSH1	1	*LOC_Os01g62920.1*	BEL1-type homeobox	[1]
OsGRF4/PT2	2	*LOC_Os02g47280.1*	Growth-regulating factor	[2]
OsSh1	3	*LOC_Os03g44710.1*	YABBY transcription factor	[3]
OsNPC1	3	*LOC_Os03g61130.1*	Phospholipase C1	[4]
sh4/*SHA1*	4	*LOC_Os04g57530.1*	Myb3 transcription factor	[5,6]
SHAT	4	*LOC_Os04g55560.2*	AP2 transcription factor	[7]
OsLG1	4	*LOC_Os04g56170.1*	SQUAMOSA promoter-binding protein	[8,9]
SH5	5	*LOC_Os05g38120.1*	BEL1-type homeobox	[10]
sh-h/*OsCPL1*	7	*LOC_Os07g10690.1*	CTD phosphatase	[11]
SSH1	7	*LOC_Os07g13170.1*	AP2 transcription factor	[12]

Supplementary Table 9. Contents of phytohormones (ng/g) between abscission layer formation (ALF) and abscission layer degradation (ALD) tissues in Chinese wild rice. Error bars indicate the standard error of the mean of the three independent experiments. Asterisks (*) indicate values that differ significantly according to Tukey's test ($P < 0.05$).

Phytohormones	Category	ALF	ALD
Abscisic acid	ABA	79.5667±17.8646	906.6667±169.9098*
ABA-glucosyl ester	ABA	17.6667±1.4572	139±29.0517*
1-Aminocyclopropanecarboxylic acid	ETH	109±5.2915	152±13.1149*
cis-Zeatin	CK	0.182±0.033	0.2757±0.0282*
Dihydrozeatin-7-Glucoside	CK	0.489±0.1037	4.2±2.5247
Dihydrozeatin	CK	0±0	0.2177±0.377
trans-Zeatin	CK	0.6427±0.0636	1.6793±0.6311
trans-Zeatin riboside	CK	0.52±0.164	1.5613±0.5313*
N6-isopentenyladenine	CK	0.1807±0.0431	0.3923±0.0335*
N6-isopentenyladenosine	CK	0.2737±0.0496	0.3583±0.1334
Gibberellin A3	GA	0.4413±0.3823	0.56±0.9699
Gibberellin A4	GA	1.2133±1.3146	0±0
Gibberellin A7	GA	0.273±0.1495	0.1196±0.0709
Gibberellin A9	GA	2.0833±0.3099*	0±0
Gibberellin A15	GA	0.2667±0.1334	0.3433±0.5861
Gibberellin A19	GA	5.0633±0.6573*	0.98±1.6974
Gibberellin A24	GA	1.3367±1.1853	0.28±0.485
Gibberellin A53	GA	3.0933±1.6407	2.13±0.3704
Indole-3-acetic acid	Auxin	3.94±1.0553	121.0333±36.3704*
1-*O*-indol-3-ylacetylglucose	Auxin	0±0	33.0333±4.9238*
Indole-3-carboxylic acid	Auxin	3.1333±0.5829	12.2767±3.6382*
Indole-3-carboxaldehyde	Auxin	9.7233±0.9063	12.5±5.5669
Methyl indole-3-acetate	Auxin	2.27±0.6528	6.9±2.0219*
3-Indolepropionic acid	Auxin	2.3333±0.5853	1.6467±0.5829
Dihydrojasmonic acid	JA	2.1333±0.2715	2.9467±1.0228
Jasmonic acid	JA	267±67.5796*	93.6±16.4037
Jasmonoyl-L-isoleucine	JA	134±4	129.9±37.5237
Methyl jasmonate	JA	0.722±0.2413*	0.1857±0.0427
cis(+)-12-oxophytodienoic acid	JA	136±17.0587	141±44.3058
Salicylic acid	SA	131.6667±23.8607	311±14.7309*
Salicylic acid 2-*O*-β-Glucoside	SA	2633.3333±303.5347	5656.6667±190.0877*

Supplementary Table 10. Expression level of genes in *Zizania latifolia* phytocassane gene cluster. ALF, abscission layer formation; ALD, abscission layer degradation.

**GeneName**	**Zlat_ID**	**Chr**	**Start**	**End**	**ALD-1**	**ALD-2**	**ALD-3**	**ALF-1**	**ALF-2**	**ALF-3**	**Leaf-1**	**Leaf-2**	**Leaf-3**	**Stem-1**	**Stem-2**	**Stem-3**
CYP76M5	Zla08G012880.1	Chr8	22538924	22540429	0.1211	0.0696	0.4592	0.2497	0.1903	0.1261	0.0547	0.1336	0.0529	0	0	0
CYP76M5	Zla08G012900.1	Chr8	22548633	22550144	0.5784	0.3715	0.6765	2.3286	3.2158	4.8435	0.1751	0	0.0488	0	0	0
KSL11	Zla08G012910.1	Chr8	22551904	22554144	0.1576	0.2362	0.4211	0.9582	1.338	0.8598	0	0	0.1186	0	0	0
CPS2	Zla08G012920.1	Chr8	22581285	22585982	0.4715	1.2934	0.604	4.9568	3.3705	1.853	0.1921	0.2442	0.4375	0	0	0
CYP76M8	Zla08G012930.1	Chr8	22592610	22594109	2.0331	5.9386	3.6573	13.1644	7.8396	3.7434	2.6255	1.5049	2.1205	0	0	0
KSL7	Zla08G012940.1	Chr8	22603752	22609836	5.0957	8.2151	5.7542	6.815	5.0724	3.092	2.3932	1.8505	3.2966	0.048	0	0.2742
CYP71Z6	Zla08G012960.1	Chr8	22619982	22621864	2.655	3.399	2.07	33.5623	33.5402	48.4151	2.2431	1.4547	0.7932	0	0	0
CYP76M7	Zla10G007850.1	Chr10	5327515	5329086	5.9354	7.1667	7.0553	11.5648	11.8023	7.157	0.987	0.6297	0.9804	0	0.1983	0
KSL5	Zla10G007880.1	Chr10	5341528	5346056	0.6015	0.7743	0.6623	2.18	2.1047	1.7305	0.2802	0.0865	0.2212	0.4069	0.2482	0.0352
KSL6	Zla10G007890.1	Chr10	5347570	5352229	4.2896	3.9419	4.3589	7.7368	9.1073	7.6731	3.2515	3.9011	4.6159	5.5705	4.2678	5.0595
CYP76M6	Zla10G007900.1	Chr10	5359473	5360972	0.084	0	0.0358	2.1724	1.7432	0.5503	0	0	0	0	0	0

Supplementary Table 11. Primers used for qRT-PCR of selected seed shattering genes

**Gene name**	**Primer**	**Sequence (5'- 3')**
Actin	Forward primer	AGAGCAGAGGCATTCCAAGT
	Reverse primer	ACTAACCGGCCACGTGTATT
ZlqSH1a	Forward primer	AACACCACTGCTTCTCAGGG
	Reverse primer	ATGCAATGTTGTGCTGCGAG
ZlqSH1b	Forward primer	CAACAACGCTGCTTCTCAGG
	Reverse primer	CAATGTTGTGCAGCGAGGAC
ZlSHATa	Forward primer	TCGACCAGAACTCGAAGCAT
	Reverse primer	CGAATTGGTGATGCCTGGGA
ZlSHATb	Forward primer	ACCCACCTAGATGACGACCA
	Reverse primer	GTGACACACACCTCCTCTCG
ZlLG1a	Forward primer	GAACGTACCATCTGCCAACT
	Reverse primer	CCGTCCTGATCCATGATTGGAA
ZlLG1b	Forward primer	GGCATCCATAGGGATCAGCA
	Reverse primer	TGAGGTAGTGGGAGCTGGTA
ZlSH5a	Forward primer	TGCTCCCTTTGCTTCCGTAG
	Reverse primer	TGCCACATCTTCTTTGCCGA
ZlSH5b	Forward primer	CGAGCTCATTGGCACTGGTA
	Reverse primer	TCAGCAATGTAAGCCCGGTT

Supplementary References

[1] Konishi, S. et al. An SNP caused loss of seed shattering during rice domestication. *Science* **312**, 1392–1396 (2006).

[2] Sun, P. et al. *OsGRF4* controls grain shape, panicle length and seed shattering in rice. *J. Integr. Plant Biol*. **58**, 836–847 (2016).

[3] Lin, Z. et al. Parallel domestication of the Shattering1 genes in cereals. *Nat. Genet*. **44**, 720–724 (2012).

[4] Cao, H. et al. Non-specific phospholipase C1 affects silicon distribution and mechanical strength in stem nodes of rice. *Plant J*. **86**, 308–321 (2016).

[5] Li, C. et al. Rice domestication by reducing shattering. *Science* **311**, 1936–1939 (2006).

[6] Lin, Z. et al. Origin of seed shattering in rice (*Oryza sativa L.*). *Planta* **226**, 11–20 (2007).

[7] Zhou, Y. et al. Genetic control of seed shattering in rice by the APETALA2 transcription factor SHATTERING ABORTION1. *Plant Cell* **24**, 1034–1048 (2012).

[8] Ishii, T. et al. *OsLG1* regulates a closed panicle trait in domesticated rice. *Nat. Genet*. **45**, 462–465 (2013).

[9] Lee, J. et al. Mutations in the rice liguleless gene result in a complete loss of the auricle, ligule, and laminar joint. *Plant Mol. Biol*. **65**, 487–499 (2007).

[10] Yoon, J. et al. The BEL1-type homeobox gene *SH5* induces seed shattering by enhancing abscission-zone development and inhibiting lignin biosynthesis. *Plant J*. **79**, 717–728 (2014).

[11] Ji, H. et al. Inactivation of the CTD phosphatase-like gene *OsCPL1* enhances the development of the abscission layer and seed shattering in rice. *Plant J*. **61**, 96–106 (2010).

[12] Jiang, L. et al. The APETALA2-Like transcription factor SUPERNUMERA RY BRACT controls rice seed shattering and seed size. *Plant Cell* **31**, 17–36 (2019).
