# Supplementary material for: Chromosome-level genome assembly of Zizania latifolia provides insights into its seed shattering and phytocassane biosynthesis
Source: Commun Biol. 2022 Jan 11;5:36. doi: 10.1038/s42003-021-02993-3 (PMC8752815; doi:10.1038/s42003-021-02993-3)
Supplement: Supplementary file 2 — Description of Additional Supplementary Files [file 42003_2021_2993_MOESM2_ESM.pdf]

Description of Additional Supplementary Files

File name: Supplementary Data 1

Description: Functional annotation of protein-coding genes in the Chinese wild rice genome.

File name: Supplementary Data 2

Description: Positive selection genes in *Zizania latifolia*.

File name: Supplementary Data 3

Description: Candidate genes for seed shattering in rice (*Oryza sativa*) and Chinese wild rice (*Zizania latifolia*).

File name: Supplementary Data 4

Description: Differentially expressed genes between abscission layer formation (ALF) and abscission layer degradation (ALD) tissues in Chinese wild rice.
