# Supplementary material for: Chromosome-level genome assembly of Zizania latifolia provides insights into its seed shattering and phytocassane biosynthesis
Source: Commun Biol. 2022 Jan 11;5:36. doi: 10.1038/s42003-021-02993-3 (PMC8752815; doi:10.1038/s42003-021-02993-3)
Supplement: Supplementary file 7 — Reporting Summary [file 42003_2021_2993_MOESM7_ESM.pdf]

nature portfolio

Corresponding author(s): *Double-blind peer review submissions: write DBPR and your manuscript number here instead of author names.*

Last updated by author(s): *YYYY-MM-DD*

# Reporting Summary

Nature Portfolio wishes to improve the reproducibility of the work that we publish. This form provides structure for consistency and transparency in reporting. For further information on Nature Portfolio policies, see our Editorial Policies and the Editorial Policy Checklist.

## Statistics

For all statistical analyses, confirm that the following items are present in the figure legend, table legend, main text, or Methods section.

| n/a | Confirmed | |
|---|---|---|
| ☐ | ☒ | The exact sample size (*n*) for each experimental group/condition, given as a discrete number and unit of measurement |
| ☐ | ☒ | A statement on whether measurements were taken from distinct samples or whether the same sample was measured repeatedly |
| ☐ | ☒ | The statistical test(s) used AND whether they are one- or two-sided<br>*Only common tests should be described solely by name; describe more complex techniques in the Methods section.* |
| ☐ | ☒ | A description of all covariates tested |
| ☐ | ☒ | A description of any assumptions or corrections, such as tests of normality and adjustment for multiple comparisons |
| ☐ | ☒ | A full description of the statistical parameters including central tendency (e.g. means) or other basic estimates (e.g. regression coefficient) AND variation (e.g. standard deviation) or associated estimates of uncertainty (e.g. confidence intervals) |
| ☐ | ☒ | For null hypothesis testing, the test statistic (e.g. *F*, *t*, *r*) with confidence intervals, effect sizes, degrees of freedom and *P* value noted<br>*Give P values as exact values whenever suitable.* |
| ☐ | ☒ | For Bayesian analysis, information on the choice of priors and Markov chain Monte Carlo settings |
| ☐ | ☒ | For hierarchical and complex designs, identification of the appropriate level for tests and full reporting of outcomes |
| ☐ | ☒ | Estimates of effect sizes (e.g. Cohen's *d*, Pearson's *r*), indicating how they were calculated |

*Our web collection on statistics for biologists contains articles on many of the points above.*

## Software and code

Policy information about availability of computer code

| | |
|---|---|
| Data collection | To determine whether the sequencing data were contaminated, we randomly selected 2,000 single end reads and compared them with those in the Nucleotide Sequence Database by BLAST; there were no contaminated sequences. For Illumina sequencing, a paired-end library with an insert size of 350bp was sequenced using the Illumina HiSeq X Ten platform (Illumina, San Diego, CA, USA) with a 150 nt layout, according to the manufacturer instructions. |
| Data analysis | Genome assembly<br>Nanopore third-generation sequencing data were corrected using Canu45; the SMARTdenovo software (https://hpc.ilri.cgiar.org/smartdenovo-software) was used to assemble the corrected data. The Racon46 (https://bioinformaticshome.com/tools/wga/descriptions/Racon.html) and Pilon47 software were used to perform three rounds of correction of the third-generation sequencing data and second-generation data, respectively.<br>The Burrows–Wheeler Alignment software32 was used to compare the short sequences obtained from Illumina sequencing with the reference genome in this study, and the integrity of the assembled genome was evaluated through statistical comparisons. The CEGMA v2.533 database containing 458 conserved eukaryotic core genes was used to evaluate the integrity of the final genome assembly. The embryophyta database in OrthoDB v10s (containing 1,614 conserved core genes) and BUSCO v4.048 were used to evaluate the integrity of the genome assembly. Additionally, the LAI value was used to judge the assembly quality based on repetitive genomic regions34.<br><br>Hi-C analysis and pseudo-chromosome construction<br>Fresh young leaves collected from Chinese wild rice Huai'an plants were fixed with 1% formaldehyde. Hi-C fragment libraries were constructed using 300- to 700-bp inserts49. The low-quality reads and adapter sequences of raw reads were removed to obtain clean data. Notably, only uniquely aligned paired reads with a mapping quality of >20 were used for further analysis. Before chromosome assembly, we performed a preassembly for error correction of scaffolds, which required the splitting of scaffolds into 50-kb segments. Hi-C data were then mapped to these segments using the Burrows–Wheeler Alignment software. The uniquely mapped data were retained to perform assembly using LACHESIS50. |



March 2021

Repetitive sequence and gene annotation
Using the LTR_FINDER51 and RepeatScout52 software, we constructed a genome repetitive-sequence database based on ab initio prediction and structure prediction. The database was classified with PASTEClassifier53 and then combined with the Repbase database54 as the final repeat-sequence database. We then used RepeatMasker55 for repetitive-sequence prediction of the genome. Default parameters were used for LTR_FINDER, RepeatScout, and PASTEClassifier, and the '-nolow -no_is -norna -engine wublast' parameter was used for RepeatMasker. Additionally, we used the EDTA (v1.9.7) software to generate TE annotations56.

Gene-structure prediction was performed for the Z. latifolia genome using ab initio prediction, prediction based on homologous species, and prediction based on Unigene analysis. The prediction results were integrated using EVM v1.1.157. First, we used Genscan58, Augustus v2.459, GlimmerHMM v3.0.460, GeneID v1.461, and SNAP62 for ab initio prediction. Second, we used GeMoMa v1.3.163,64 for prediction based on homologous species. Stringtie v1.2.365 and Hisat v2.0.466, and GeneMarkS-T v5.167 and TransDecoder v2.0, were used for assembly and gene prediction, respectively. We sequenced the mixed RNA library generated from the root, stem, leaf, leaf sheath, male and female florets, seed, and a whole un-emerged panicle for transcriptome-based predictions. Additionally, the RNA-seq reads were assembled into transcripts using Trinity v2.1.168, and PASA v2.0.269 was used to predict the Unigene based on RNA-seq reads.

Noncoding RNAs include miRNA, rRNA, tRNA, and other RNAs with known functions. Blastn was used for genome-wide alignment based on the Rfam database70 to identify miRNAs and rRNAs, and tRNAscan-SE71 was used to identify tRNAs. The predicted protein sequences were used to search for homologous gene sequences through GenBlastA72 alignment, and GeneWise73 was then used to search for premature stop codons and frameshift mutations that resulted in pseudogenes. For GenBlastA, an e-value of 1 10−5 was used; all other parameters were set to default. Additionally, default parameters were used for GeneWise. BLAST v2.2.3174 alignment (e-value: 1 10−5) was performed between the predicted gene sequence and the Non-Redundant Protein Sequence Database75, EuKaryotic Orthologous Groups76, Gene Ontology77, KEGG78, and TrEMBL79 functional databases.

Gene families and phylogenetic analysis
Orthofinder v2.480 was used to classify the protein sequences of nine gramineous plants (B. distachyon, H. vulgare, L. perrieri, O. brachyantha, O. sativa, S. bicolor, S. italica, Z. latifolia, and Z. mays) and one dicotyledon (A. thaliana) into families. The PANTHER V15 database81 was used for annotation of the gene families obtained. IQ-TREE v1.6.1182 was used to construct a phylogenetic tree from 1,371 single-copy protein sequences. Specifically, MAFFT v7.205 (https://mafft.cbrc.jp/alignment/software/) was used to align each single-copy gene family sequence, and the PAL2NAL v14 program83 was then used to convert the protein alignment to codon alignment. We then used Gblocks v0.91b (parameter: -b5=h)84 to remove regions with large differences or poor sequence alignment. Finally, the aligned gene family sequences of each species were connected end-to-end to obtain a super-gene alignment. The model testing tool ModelFinder85, included with IQ-TREE (http://www.iqtree.org/), was used for model selection, with the best model identified as GTR+F+I+G4. Using this model, we applied the maximum-likelihood method to construct a phylogenetic tree, with the number of bootstraps set to 1,000. The outgroup of the obtained phylogenetic tree was set as A. thaliana, which gave a rooted tree, and the MCMCTREE package included in the PAML v4.9i software86 was then used to calculate divergence times. The final phylogenetic tree with divergence times was displayed graphically using MCMCTreeR v1.187. CAFE v4.288 was used with the phylogenetic tree with divergence times and genes (after clustering into families) to estimate the number of gene family members of an ancestor from each branch through birth-death models, predicting the contraction and expansion of a gene family from each species relative to that of its ancestor. Significant expansion or contraction was defined as family-wide p-values and viterbi p-values (both <0.05).

We used the CodeML module in PAML for positive-selection analysis. First, we obtained single-copy gene families common among B. distachyon, H. vulgare, L. perrieri, O. brachyantha, O. sativa, and Z. latifolia, followed by MAFFT (parameters: --localpair --maxiterate 1000) alignment of the protein sequences of each gene family and conversion to the codon alignment sequence using PAL2NAL. Finally, CodeML was used to perform likelihood ratio tests of model A and the null model using the 'chi2' program in PAML based on the branch-site model. An empirical Bayes method was used to obtain the posterior probability of being considered a positively selected site (>0.95 is usually considered a significantly positively selected site).

Collinearity and WGD analyses
We used Diamond v0.9.29.13089 to compare the protein sequences of O. sativa and Z. latifolia (C-score>0.5; e-value<1×10−5). Subsequently, we identified the collinear blocks between the genomes of O. sativa and Z. latifolia using MCScanX90. Finally, based on the distribution of the Ks paralogous genes, we calculated the WGD events using the WGD software91.

Identification of seed-shattering genes in Chinese wild rice
Genes related to seed shattering in O. sativa were obtained by querying the gene name on the website of the China Rice Data Centre (https://www.ricedata.cn/). Seed-shattering genes in Z. latifolia were obtained by comparing similar genes in O. sativa with the genome sequences of Z. latifolia in this study. The e-value of the sequence-alignment results was set to <1 10−10. MCScanX was used for collinearity analysis of candidate genes.

For manuscripts utilizing custom algorithms or software that are central to the research but not yet described in published literature, software must be made available to editors and reviewers. We strongly encourage code deposition in a community repository (e.g. GitHub). See the Nature Portfolio guidelines for submitting code & software for further information.

# Data

Policy information about availability of data
All manuscripts must include a data availability statement. This statement should provide the following information, where applicable:
- Accession codes, unique identifiers, or web links for publicly available datasets
- A description of any restrictions on data availability
- For clinical datasets or third party data, please ensure that the statement adheres to our policy

Data availability
The raw genome and transcriptome sequencing data generated for this work are accessible via the NCBI Sequence Read Archive under accession number PRJNA719466. The whole genome sequence data reported in this paper have been deposited in the Genome Warehouse in the National Genomics Data Center, Beijing Institute of Genomics, Chinese Academy of Sciences/China National Center for Bioinformation, under accession number GWHBFHI00000000, which is publicly accessible at https://ngdc.cncb.ac.cn/gwh.

# Field-specific reporting

Please select the one below that is the best fit for your research. If you are not sure, read the appropriate sections before making your selection.

[x] Life sciences [ ] Behavioural & social sciences [ ] Ecological, evolutionary & environmental sciences

For a reference copy of the document with all sections, see nature.com/documents/nr-reporting-summary-flat.pdf

# Life sciences study design

All studies must disclose on these points even when the disclosure is negative.

| | |
|---|---|
| Sample size | The sampling site is located in Baimahu Village, Jinhu County, Huai'an City, Jiangsu Province (33°11′9″ N; 119°9′37″ E)2. Owing to the relatively closed geographical environment, Chinese wild rice in this region is highly homozygous. Leaf samples from Chinese wild rice Huai'an were collected for sequencing. |
| Data exclusions | After DNA sequencing, reads with adapters, low-quality reads, and reads of <2,000nt were filtered. |
| Replication | In Supplementary Fig. 17 and Supplementary Table 9, we used n = 3 biologically independent samples. |
| Randomization | This item is not involved. |
| Blinding | This item is not involved. |

# Reporting for specific materials, systems and methods

We require information from authors about some types of materials, experimental systems and methods used in many studies. Here, indicate whether each material, system or method listed is relevant to your study. If you are not sure if a list item applies to your research, read the appropriate section before selecting a response.

## Materials & experimental systems

| n/a | Involved in the study |
|---|---|
| [x] | [ ] Antibodies |
| [x] | [ ] Eukaryotic cell lines |
| [x] | [ ] Palaeontology and archaeology |
| [x] | [ ] Animals and other organisms |
| [x] | [ ] Human research participants |
| [x] | [ ] Clinical data |
| [x] | [ ] Dual use research of concern |

## Methods

| n/a | Involved in the study |
|---|---|
| [x] | [ ] ChIP-seq |
| [x] | [ ] Flow cytometry |
| [x] | [ ] MRI-based neuroimaging |
